# Promiscuous signaling by a regulatory system unique to the pandemic PMEN1 pneumococcal lineage

**DOI:** 10.1371/journal.ppat.1006339

**Published:** 2017-05-18

**Authors:** Anagha Kadam, Rory A. Eutsey, Jason Rosch, Xinyu Miao, Mark Longwell, Wenjie Xu, Carol A. Woolford, Todd Hillman, Anfal Shakir Motib, Hasan Yesilkaya, Aaron P. Mitchell, N. Luisa Hiller

**Affiliations:** 1 Department of Biological Sciences, Carnegie Mellon University, Pittsburgh, Pennsylvania, United States of America; 2 Infectious Diseases, St. Jude Children’s Research Hospital, Memphis, Tennessee, United States of America; 3 Center of Excellence in Biofilm Research, Allegheny Health Network, Pittsburgh, Pennsylvania, United States of America; 4 Pittsburgh Ear Associates, Allegheny General Hospital, Pittsburgh, Pennsylvania, United States of America; 5 Department of Infection, Immunity & Inflammation, University of Leicester, Leicester, United Kingdom; The University of Alabama at Birmingham, UNITED STATES

## Abstract

*Streptococcus pneumoniae* (pneumococcus) is a leading cause of death and disease in children and elderly. Genetic variability among isolates from this species is high. These differences, often the product of gene loss or gene acquisition via horizontal gene transfer, can endow strains with new molecular pathways, diverse phenotypes, and ecological advantages. PMEN1 is a widespread and multidrug-resistant pneumococcal lineage. Using comparative genomics we have determined that a regulator-peptide signal transduction system, TprA2/PhrA2, was acquired by a PMEN1 ancestor and is encoded by the vast majority of strains in this lineage. We show that TprA2 is a negative regulator of a PMEN1-specific gene encoding a lanthionine-containing peptide (*lcpA*). The activity of TprA2 is modulated by its cognate peptide, PhrA2. Expression of *phrA2* is density-dependent and its C-terminus relieves TprA2-mediated inhibition leading to expression of *lcpA*. In the pneumococcal mouse model with intranasal inoculation, TprA2 had no effect on nasopharyngeal colonization but was associated with decreased lung disease via its control of *lcpA* levels. Furthermore, the TprA2/PhrA2 system has integrated into the pneumococcal regulatory circuitry, as PhrA2 activates TprA/PhrA, a second regulator-peptide signal transduction system widespread among pneumococci. Extracellular PhrA2 can release TprA-mediated inhibition, activating expression of TprA-repressed genes in both PMEN1 cells as well as another pneumococcal lineage. Acquisition of TprA2/PhrA2 has provided PMEN1 isolates with a mechanism to promote commensalism over dissemination and control inter-strain gene regulation.

## Author summary

*Streptococcus pneumoniae* (pneumococcus), an important human pathogen, exhibits a dual lifestyle featuring asymptomatic colonization of the host on the one hand as well as infliction of severe local and systemic disease on the other. In pneumococcal strains, differences in gene possession often lead to varied phenotypic outcomes. Epidemiologically, pandemic strains of the PMEN1 lineage show high prevalence in disease as well as carriage, posing an interesting question on the composition and function of the genomic toolkit that leads to their widespread success. Here, we characterize TprA2/PhrA2 sensory system, a genomic region acquired exclusively by the PMEN1 strains. The system consists of a regulator-peptide pair that was horizontally acquired into PMEN1 along with its regulatory circuitry. The regulatory peptide PhrA2 is receptive to cell density of PMEN1 cells and is an example of elegant communication signaling between bacterial cells. The regulatory influence of PhrA2 extends beyond PMEN1 cells such that it controls genes of a widespread signaling system and virulence regulon in non-PMEN1 strains. This work contributes to the knowledge of peptide-communication signals in pneumococcus and further adds a novel mechanism by which an ecologically successful linage may modify the transcriptomic and functional landscape of a multi-strain pneumococcal community.

## Introduction

*Streptococcus pneumoniae* (pneumococcus) is one of the most important community acquired human pathogens, and is responsible for an estimated 850,000 deaths annually in children under the age of 5[1]. Pneumococcus colonizes the nasopharynx of young children at very high rates, and is asymptomatic in most cases [2,3]. However, it can also disseminate from the nasopharynx into tissues leading to diseases such as otitis media, pneumonia, bacteremia, meningitis, and inflammation of the heart [4–6]. The pneumococcal molecules responsible for this transition from a commensal to a pathogen are not well understood. Here we characterize a novel quorum sensing (QS) system (TprA2/PhrA2) that limits pneumococcal disease, without affecting nasopharyngeal colonization.

At the genomic level, there is extensive diversity among pneumococccal lineages. These genomic variations contribute to the differences in colonization and virulence potential [7]. Only half of the pangenome is shared across all strains (core set), while the other half is unevenly distributed amongst isolates [8,9]. The Pneumococcal Molecular Epidemiology Network (PMEN) has grouped strains of multi locus sequencing type (MLST) 81 into the PMEN1 lineage (also known as Spain^23F^-1 and SPN23F) [10]. Over the past 30 years, PMEN1 has distinguished itself by its worldwide distribution, multi-drug resistant profile, and emergence of vaccine-escape strains.

Historically, the PMEN1 lineage was responsible for the Spanish epidemic of the 1980s and has since spread to North and South America, Europe, Asia, Africa, and Australia [2,10]. Most PMEN1 isolates are resistant to penicillin, chloramphenicol, and tetracycline, and many isolates have additional resistances to fluoroquinilones and macrolides [11,12]. PMEN1 isolates are predominantly of serotype 23F, but there are also capsular switches to other serotypes, some of which represent vaccine-escape isolates [13]. Further, the PMEN1 lineage has impacted the genome content of the pneumococcal population by virtue of its high frequency of DNA donation, including genes for drug-resistance, to other pneumococcal lineages [14]. The PMEN1 genome encodes an integrative conjugative element (ICESp23FST81) [13,15,16]. As described by Croucher and colleagues upon sequencing of the first PMEN1 genome, this ICE encodes drug resistance determinants, a complete lanthionine-peptide gene cluster and a regulator-peptide pair, which in this study we have identified as the TprA2/PhrA2 QS system.

Quorum sensing systems serve as a critical, decision-making process in the response of bacteria to the environment, and their ability to colonize and/or disseminate to tissues. The best characterized kind of QS machinery is the two component system, where the signal is sensed by a surface-localized histidine kinase and transferred to a cytosolic response regulator [17]. Streptococci, enterococci and bacilli have been shown to encode a second kind of QS characterized by the emerging RRNPP (Rgg/Rap/NprR/PlcR/PrgX) superfamily of transcriptional regulators and their cognate peptides [18]. In these systems, the secreted peptide is exported from the producer cell, processed, and imported into the cytosol of producing or neighboring cells, where it interacts with the RRNPP regulator [18]. RRNPP-peptide systems have been shown to regulate virulence, biofilm formation, and the production of bacteriocins [19–21].

In pneumococcus, the majority of characterized peptides signal via two component systems [17]. These peptides regulate competence and class II bacteriocin production [22,23]. The first RRNPP-peptide pair was recently characterized in the pneumococcus strain D39 [24]. It is composed of the TprA regulator and its cognate peptide PhrA. PhrA alleviates gene inhibition leading to the expression of physiologically important genes [24]. PhrA levels are repressed by glucose and activated by galactose, consistent with activity in the upper respiratory track where galactose is a major source of energy [25].

In this study we characterize the TprA2/PhrA2 QS system, a novel pneumococcal RRNPP-peptide pair, highly expressed in middle ear effusions. TprA2/PhrA2 is present almost exclusively in PMEN1 isolates where it restrains dissemination. Unlike other lineages, the PMEN1 strains encode both the TprA/PhrA and the TprA2/PhrA2 signaling systems. Extracellular PhrA2 leads to induction of TprA in PMEN1 cells as well as in D39 cells. Thus, horizontal acquisition of TprA2/PhrA2 has provided the PMEN1 lineage with a QS system and associated regulon, as well as the molecular machinery to regulate a widespread cell-cell communication system and in doing so, influence not only its own gene expression but also that of other strains.

## Results

### The genes encoding the TprA2/PhrA2 system are enriched in the PMEN1 lineage

**Fig 1:**
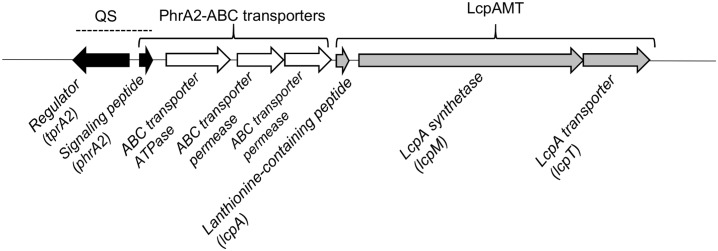
Genomic organization of TprA2/PhrA2 region in PMEN1 strains.

Genes enriched in the PMEN1 strains may provide this lineage with exclusive phenotypic properties, explaining its prevalent occurrence and rapid spread. We performed a comparative genomic screen to search for genes that are present in the majority of the PMEN1 isolates, but absent in other pneumococcal lineages. The analysis was performed on 60 pneumococcal genomes, selected to capture the diversity in the pneumococcal population (S1 Table, **labeled “To establish PMEN1 enrichment”**). We employed RAST [26] to annotate the whole genome sequences (WGS) into 125,612 coding sequences (CDSs), and organized these into 3,571 clusters of homologous sequences as previously described [27]. The screen identified a genomic region present only in the PMEN1 strains. This region encodes a transcriptional regulator (*tprA2*) on the opposite strand of a small peptide (*phrA2*) and three ABC transporters. Immediately downstream are three genes *lcpA*, *lcpM*, and *lcpT*. LcpA encodes a putative 71aa peptide with the full size weight of 7.5kDa, which we predict is a lanthionine containing peptide. Lanthionine and methyllanthionine are usually formed by the dehydration of threonines or serines, and subsequent cyclization to cysteine (*lcpA* encodes for serine, threonines, and cysteines) [28]. Cyclization is performed by lanthipeptide synthetases, of which there are four known classes [29]. The *lcpM* gene downstream of LcpA is consistent with class II synthetases (CDD score: LanM-like e-value 0e+00 [30]). Finally, the *lcpT* encodes a transporter with a C39 peptidase domain, which we predict is involved in LcpA cleavage and export (Fig 1, S2 Table).

**Fig 2:**
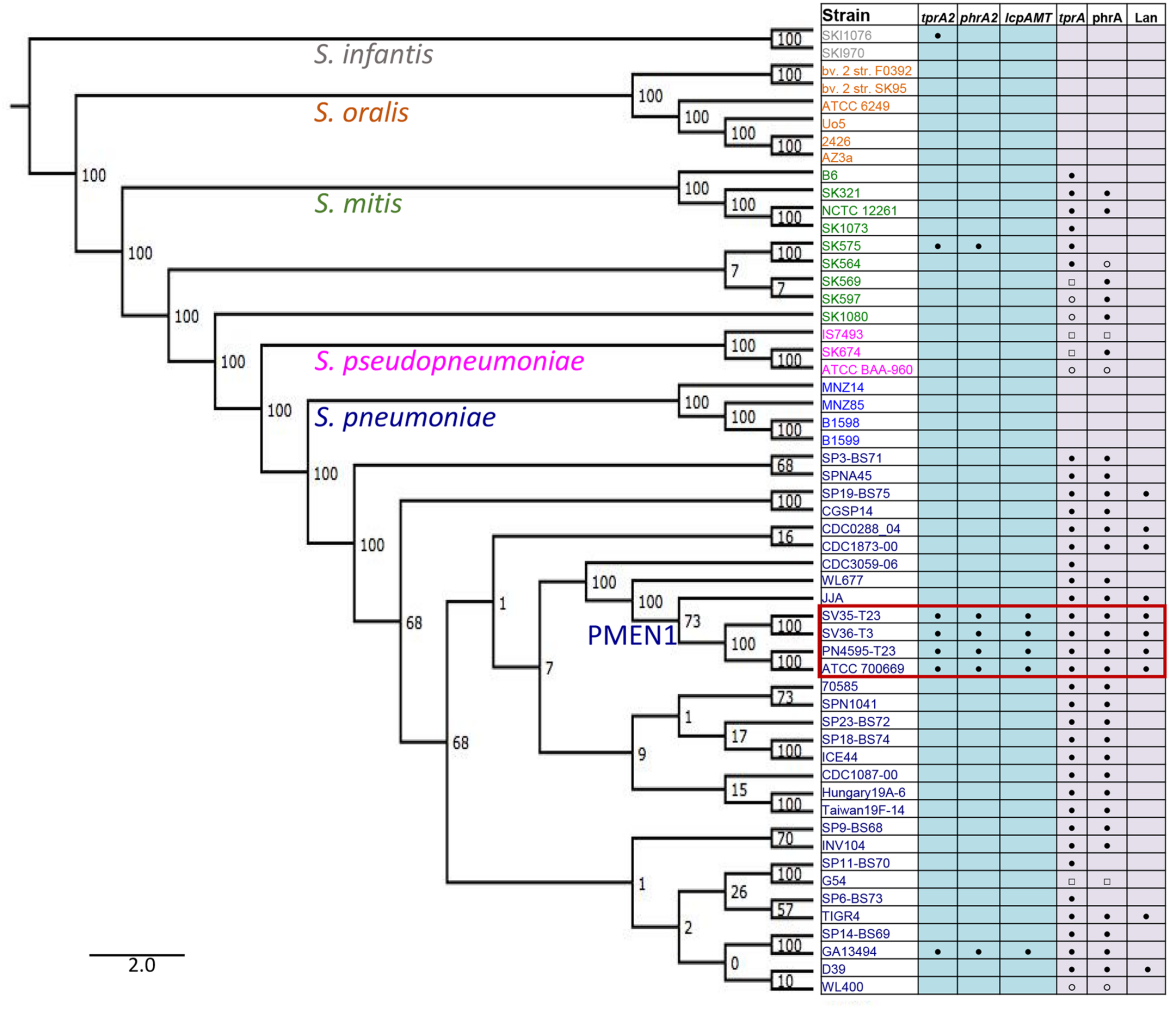
Intra- and inter-species distribution of the TprA2/PhrA2 and TprA/PhrA genomic regions. Phylogenetic analyses displaying bootstrap values on the branches. Left side: Maximum likelihood tree of streptococcal genomes generated from the core genome. Right side: Gene distribution, where blue columns display the distribution of *tprA2*, *phrA2*, and associated *lcpAMT*, and purple columns display the distribution of *tprA*, *phrA*, and downstream lantibiotic genes (seven consecutive genes, including predicted *lanA* and *lanM* labeled as Lan). Presence of the gene is marked with the following symbols: ‘●’ gene present in one copy; ‘○’ low coverage of region; ‘□’multiple copies of the gene. Red box indicates isolates from the PMEN1 lineage.

We performed a detailed assessment on the phylogenetic distribution of the QS-Lcp genes in the pneumococcus species and the *Streptococcus* genus. First, for the assessment of the distribution of TprA2 in the PMEN1 lineage, we searched for this gene in 215 PMEN1 isolates. To this end we used either polymerase chain reaction (PCR) or genomic data assembled by Croucher and colleagues [13]. The *tprA2* gene was present in 212 isolates. It was either disrupted or deleted in the genomes of strains 111 (ERS004810), 11933 (ERS005313) and HKP38 (ERS004775) (genome data was confirmed by PCR). Next, we broadened our search into the non-redundant database, which revealed that *tprA2* was present in only one strain outside the PMEN1 lineage (GA13494) [15] (Fig 2). Finally, we expanded our search for *tprA2* in related streptococcal species, specifically *S*. *pseudopneumoniae*, *S*. *mitis*, *S*. *oralis*, and *S*. *infantis* (S1 Table **labeled “Distribution with Streptococcus sp”**). We found one occurrence in *S*. *mitis* and one in *S*. *infantis*, but these species did not encode the downstream *lcpAMT* locus (Fig 2). These phylogenetic analyses demonstrate that the QS system and *lcpAMT* are present in >98% of the PMEN1 isolates and are rare outside this lineage. This distribution suggests these genes were acquired via horizontal gene transfer by a PMEN1 ancestral strain.

### QS-Lcp genes are induced and highly expressed *in vivo*

**Fig 3:**
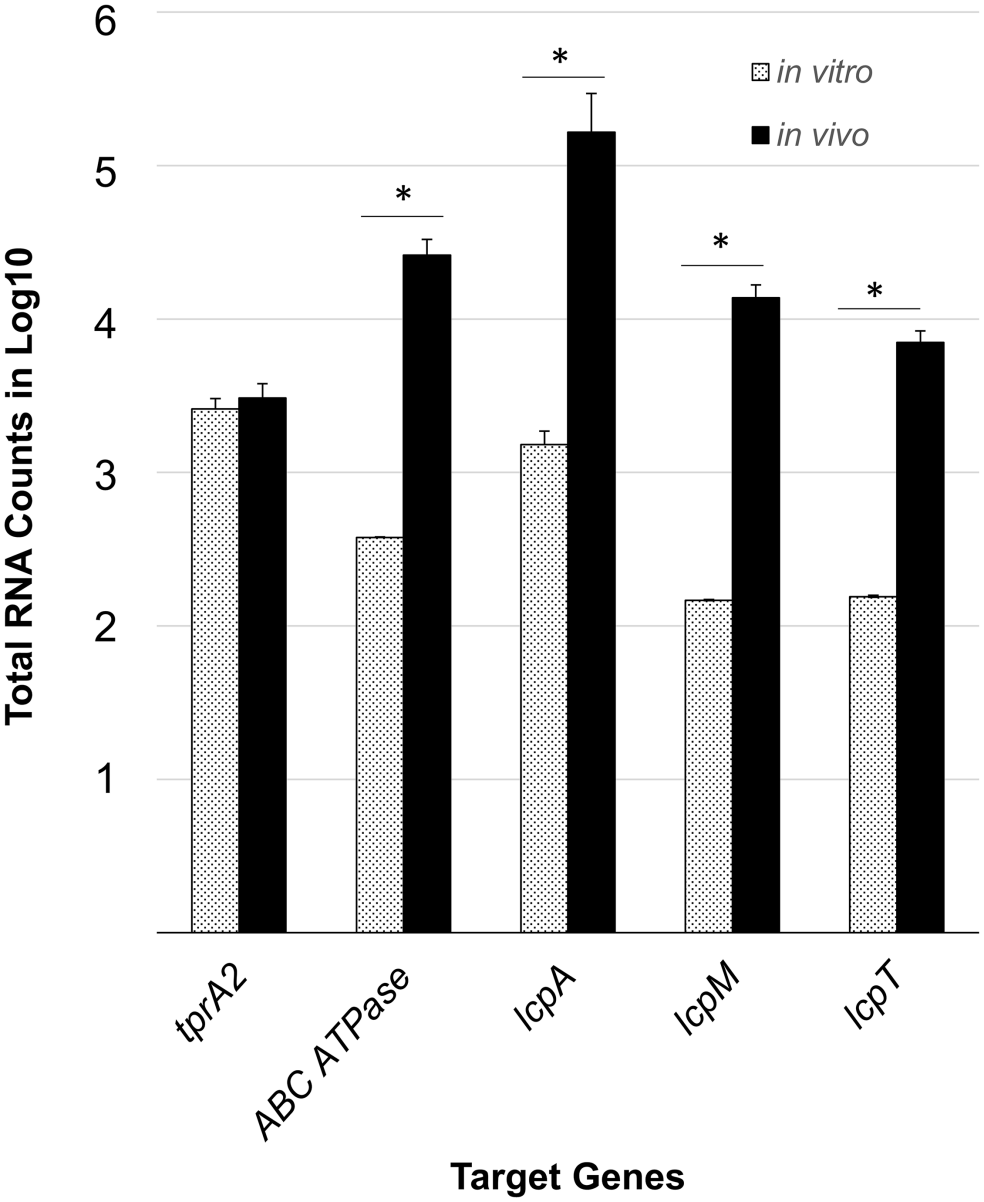
Gene expression levels of the TprA2/PhrA2 system and associated *lcpAMT* locus in chinchilla middle ear effusions and planktonic cultures. nCounter nanoString technology was used to quantify mRNA transcripts from planktonic cultures (dotted bars, n = 2) and chinchilla middle ear effusions (black bars, n = 3). Data was normalized to the geometric mean of the expression of *gyrB* and *metG* using nSolver software. The X-axis denotes the test genes assayed for gene expression. The Y-axis displays the log_10_ of the total number of transcripts for each gene averaged over biological replicates. Error bars represent the standard deviation. ‘*’ Significantly higher *in vivo* expression (*P*-value < 0.05), as determined by Student’s *t*-test.

To determine whether QS-Lcp genes are active during infection, we measured their gene expression during middle ear infection. We utilized the nCounter NanoString technology since this allows for an automated, highly sensitive enumeration of pathogen’s mRNA transcripts in the infected host tissue. Our probes capture *tprA2*, *lcpA*, *lcpM*, and *lcpT*. Further, since we were unable to design a probe for the short coding sequence of *phrA2*, we used *ABCATPase* as a proxy since it is present on the same transcript (S1 Fig). For normalization we used probes to *gyrB* and *metG*, and normalized to the geometric mean of these housekeeping genes. The PMEN1 strain PN4595-T23 [31] was inoculated transbullarly into the chinchilla. We isolated RNA from effusions of the chinchilla middle ears at 48h post-transbullar inoculation. All five genes were expressed in middle ear effusions (Fig 3). The average counts for *ABCATPase and lcpA* were comparable to those of *psaA* (56,036 counts), which has been shown to be highly expressed *in vivo* [32], consistent with high levels of QS-Lcp *in vivo*.

To evaluate whether these genes were induced in the middle ear relative to growth in rich media, we calculated the ratio of the average number of transcripts between middle ear effusions and *in vitro* planktonic cultures. The gene expression levels of *ABCATPase*, *lcpA*, *lcpM* and *lcpT* were 69, 108, 93 and 45-fold higher *in vivo* relative to planktonic cultures, respectively. From these *in vitro* and *in vivo* measurements we infer that the QS-Lcp system is both induced and highly expressed during infection.

### The expression of *phrA2* is regulated in a density-dependent manner

**Fig 4:**
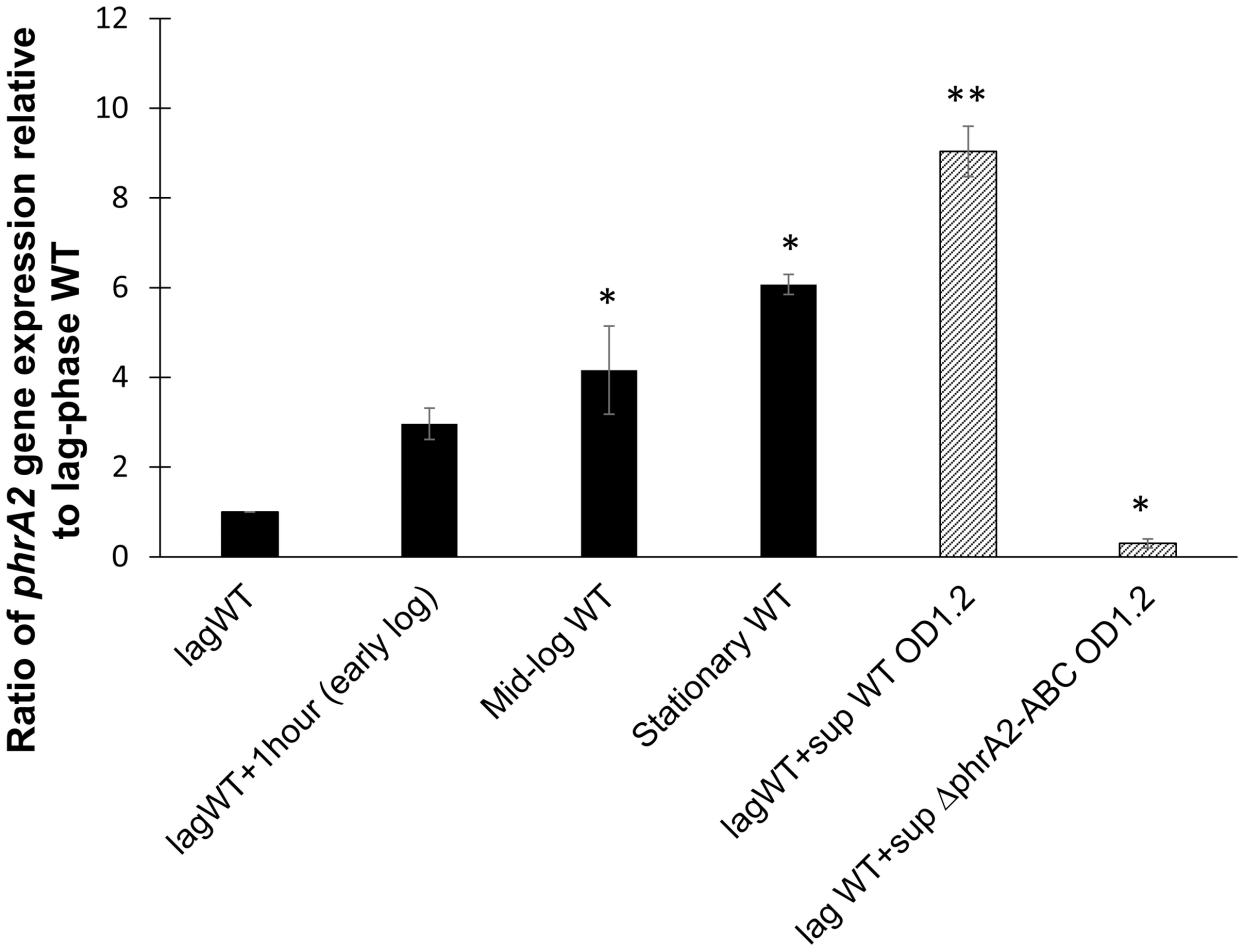
Density-dependent gene expression and extracellular secretion of *phrA2* during planktonic growth. qRT-PCR measurements of *phrA2* gene expression in PN4595-T23. The Y-axis displays expression levels as a ratio to expression in lag phase culture. The X-axis denotes culture conditions. Black bars displays density-dependent gene expression at lag phase (OD_600_0.05), early-log phase (OD_600_0.2), mid-log phase (OD_600_0.6), and stationary phase (OD_600_1.0). Striped bars display treatment by cell-free supernatants. The lag phase culture was divided into three tubes and grown for 1h in one of three ways in: original supernatant (lagWT+1hour), cell-free supernatant from a high density wild type culture (OD_600_1.2), or cell-free supernatant from a high density *ΔphrA2-ABC* culture (OD_600_1.2). 16SrRNA was used as normalization control. Error bars represent standard deviations from biological duplicate experiments. ‘**’ *P*-value<0.01 and ‘*’, *P*-value<0.05 as determined by Student’s *t*-test.

The expression of sensory peptides can be cell-density dependent (reviewed in detail in [33]. Using quantitative real time PCR (qRT-PCR) we found that *phrA2* is regulated in a density-dependent manner. Expression of *phrA2* increases at higher cell density, as observed by measuring gene expression at lag, early-log, mid-log and stationary phase (Fig 4, solid bars). Further, when a lag phase culture was left to grow for one hour, the levels of *phrA2* expression increased 3 fold. When the same culture was exposed to cell-free supernatant from a wild-type high-density culture, the levels of *phrA2* expression increased 8 fold. Yet, when it was exposed to the cell-free supernatant from a Δ*phrA2-ABC* high-density culture, the levels of *phrA2* did not increase (Fig 4, striped bars). Thus, the wild-type cells but not the Δ*phrA2-ABC* mutant, secrete a molecule that induces expression of *phrA2* in the population. These data are consistent with secretion and autoinduction of PhrA2.

### TprA2 is a negative regulator of *phrA2-ABC* and *lcpAMT*

To identify the TprA2 regulon, we compared the gene expression levels of the wild-type (WT) PMEN1 strain PN4595-T23 and the isogenic *tprA2* deletion mutant (Δ*tprA2)*, utilizing a pneumococcal gene array (S5 Table) [34]. The expression of the *phrA2-ABC* and *lcpAMT* genes were >30-fold higher in Δ*tprA2* relative to the WT strain. These results were verified, using independent biological replicates, by both qRT-PCR and nanoString technology (Table 1). These findings suggest that TprA2 is a negative regulator of these neighboring genes.

**Table 1:** Gene expression in *ΔtprA2* compared to the PN4595-T23 (WT) in planktonic cultures represented as ratio to the WT level (n = 3).

Gene ID	Target Gene	qRT-PCR		Microarray		Nanostring	
		Δ*tprA2*/WT	*P*-value	Δ*tprA2*/WT	*P*-value	Δ*tprA2*/WT	*P*-value
CGSSp4595_1262	*tprA2*	0		-19	<2.2E-16	NA	2.18E-02
CGSSp4595_1261	*phrA2*	70.7	5.54E-04	+32.6	<2.2E-16	NA	NA
CGSSp4595_1260	*ABC transporter (ATPase)*	85.6	5.52E-03	+34.7	<2.2E-16	+63.8	1.06E-02
Not annotated	*lcpA*	45.2	9.26E-04	+62.1	<2.2E-16	+71	2.25E-02
CGSSp4595_1257	*lcpM*	37.9	5.32E-03	+45.4	<2.2E-16	+57.8	1.65E-02
CGSSp4595_1256	*lcpT*	37.2	4.11E-03	+40.4	<2.2E-16	+47.3	2.12E-02

**Fig 5:**
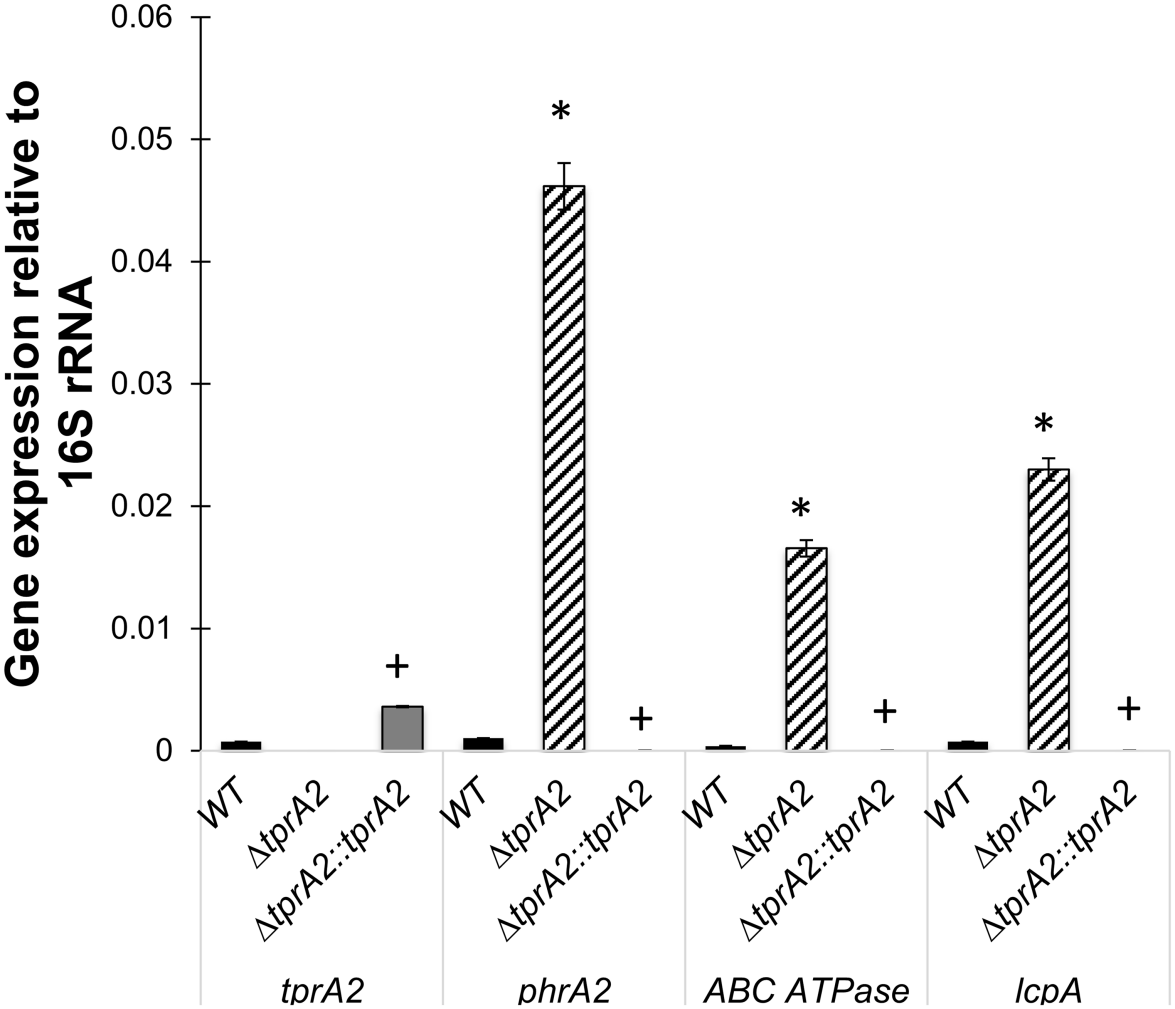
Analysis of the TprA2 regulon by comparison of gene expression levels among WT, Δ*tprA2*, and Δ*tprA2*::*tprA2* strains. qRT-PCR measurements for genes *tprA2*, *phrA2*, *ABCATPase* and *lcpA*. X-axis represents genes that were tested for expression in strains WT, *ΔtprA2* and *ΔtprA2*::*tprA2*. Y-axis denotes starting concentration of mRNA in arbitrary fluorescence units as calculated from LinRegPCR. Data was normalized to the expression of 16S rRNA. Error bars represent standard deviation for biological replicates (n = 3).‘*’ significantly different expression relative to WT (*P*-value < 0.005), ‘+’ significantly different expression relative to Δ*tprA2* (*P*-value < 0.005).

To confirm the role of TprA2, we generated a complemented strain (Δ*tprA2*::*tprA2*) where *tprA2* was inserted into the *ΔtprA2* strain at a distant chromosomal location, under the influence of the constitutive erythromycin-resistance gene promoter (*ermB*). We measured gene expression of *tprA2*, *phrA2*, *ABC transporter ATPase*, and *lcpA* in the WT, Δ*tprA2* and Δ*tprA2*::*tprA2* strains (Fig 5). The *tprA2* gene was expressed in the Δ*tprA2*::*tprA2* strain, and its expression level was higher than in the WT. Further, low levels of *phrA2*, *ABC transporter ATPase*, and *lcpA* were re-established in the complement strain. These findings strongly support our conclusion that the gene product of *tprA2* is a negative regulator of *phrA2* and *lcpAMT*.

### PhrA2 modulates the TprA2 regulon

**Fig 6:**
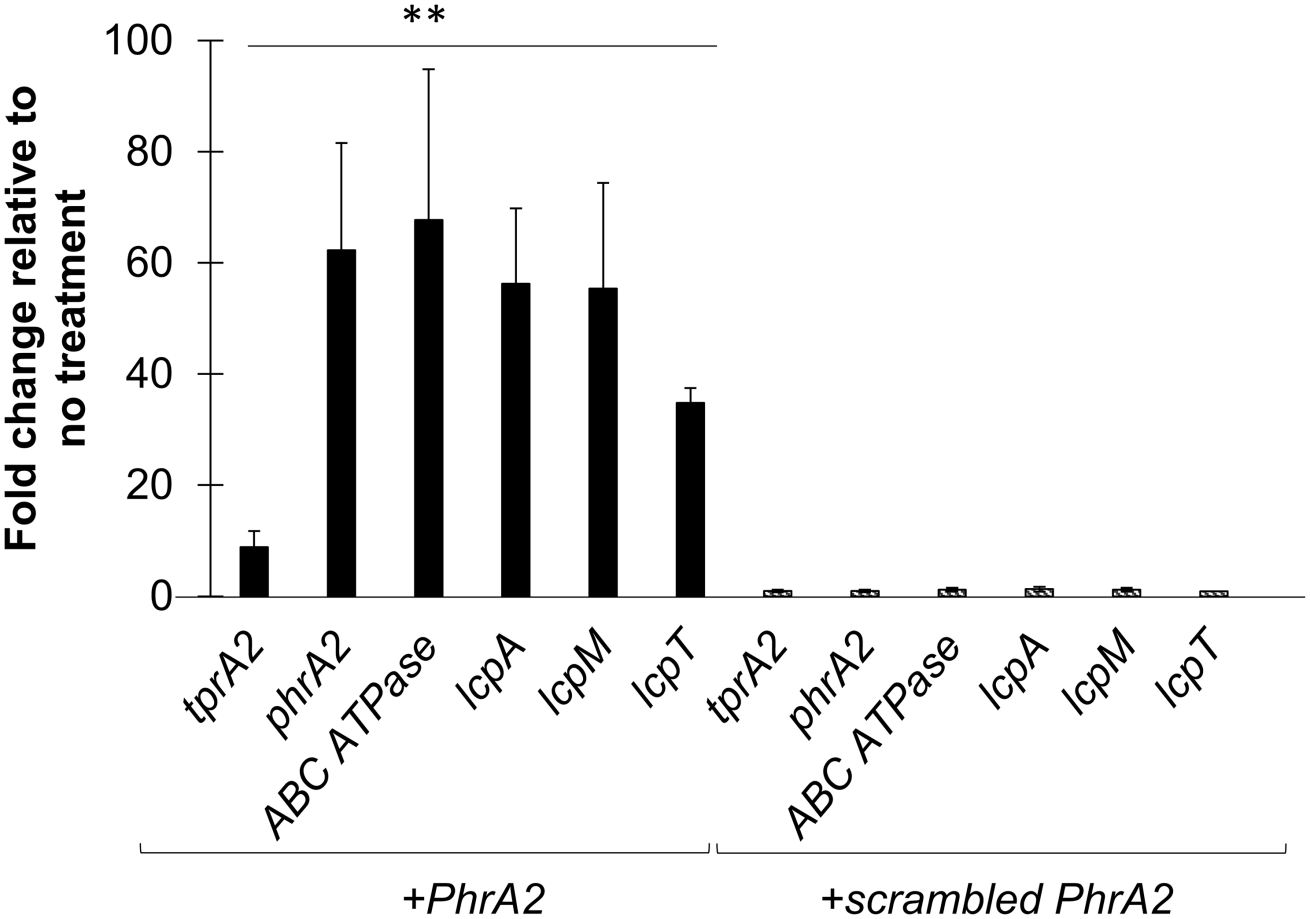
Gene expression measured by qRT-PCR of QS-Lcp genes in WT strain PN4595-T23 upon treatments. Data was normalized to 16S rRNA expression. Y-axis displays fold change in gene expression upon exposure to a peptide treatment relative to untreated control. Error bars represent standard deviations for biological replicates (n = 3). On the left, dark bars display expression from cells exposed to the PhrA2 C-terminal heptapeptide (VDLGLAD); on the right side, stripped bars display expression from cells exposed to the scrambled control peptide (DAGVLDL). “**” Statistically significant difference in gene expression after PhrA2 treatment compared to scrambled peptide (*P*-value<0.01).

The TprA2 regulator displays sequence similarity to the *Bacillus* sp. transcription factor, PlcR and to the pneumococcal TprA, which are regulated by extracellular forms of the C-terminal heptapeptides from their cognate peptides [24,35]. Given that TprA2 is part of the PlcR family, we hypothesized that the C-terminal heptapeptide of PhrA2 would encompass a functional peptide capable of influencing TprA2 activity. Thus, we utilized synthetic peptides corresponding to the seven terminal residues of PhrA2 (sequence: VDLGLAD) and a scrambled control (sequence: DAGVLDL). Addition of the PhrA2 peptide, but not the scrambled peptide to planktonic culture led to a significant increase in expression levels of *tprA2*, *phrA2*, *ABC transporter ATPase* and adjacent *lcpAMT* genes (Fig 6). The PhrA2 peptide up-regulates its own production demonstrating autoinduction of this density-dependent system. We also observed an increase in the levels of *tprA2* suggesting that TprA2 serves as a negative regulator of its own expression.

The induction of gene expression by the synthetic peptide explains the observation that supernatant from a high-density WT culture, but not a Δ*phrA2-ABC*, can induce gene expression (Fig 4). Further, cell-free supernatant from a PhrA2 overexpressing strain increases levels of *phrA2* and *lcpA* by over 5 fold when compared to media alone (S2 Fig). These findings strongly support a model in which the *phrA2* gene product is exported.

### TprA2 regulon in the middle ear

**Fig 7:**
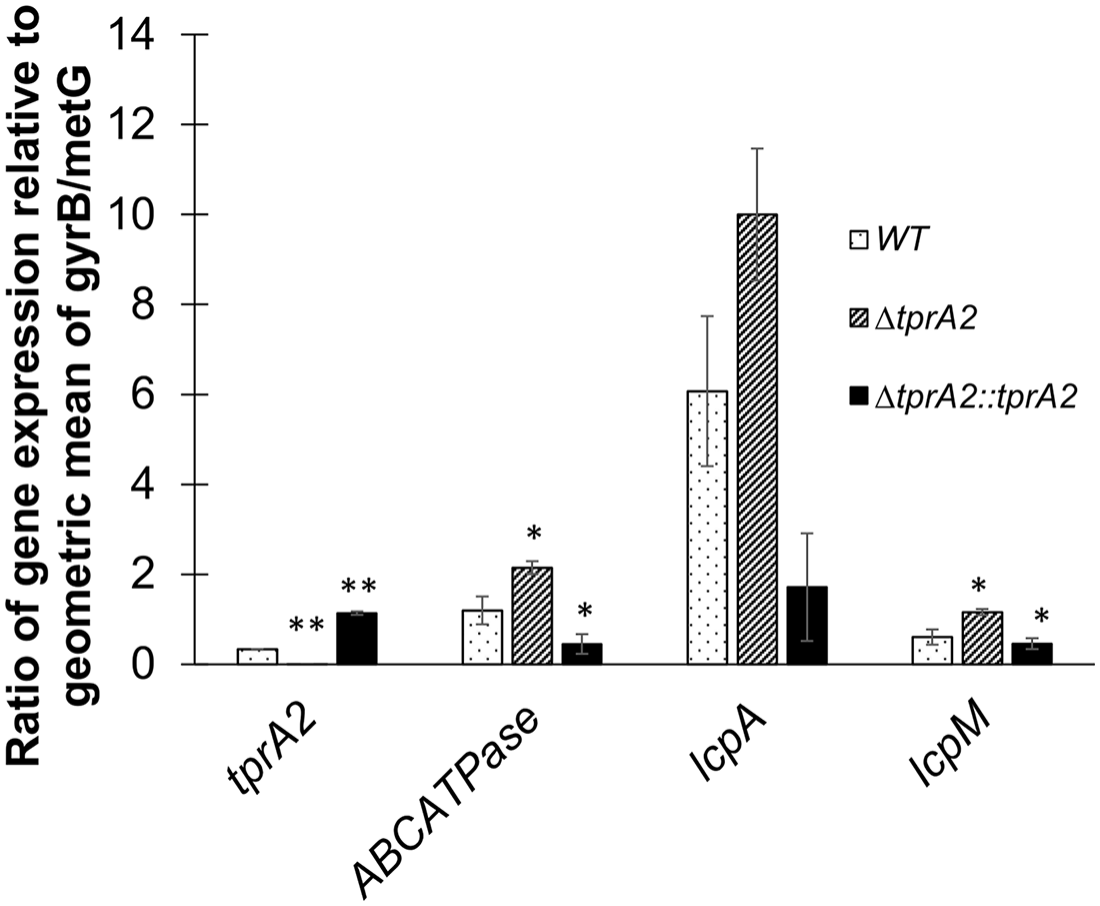
Gene expression of TprA2 regulon in the middle ear. Bars represent gene expression as measured by nCounter platform by NanoString technology on RNA extracted from middle ear effusions of chinchillas cohorts (n = 3) infected with three different strains: WT (dotted bars), Δ*tprA2* (striped bars), and *ΔtprA2*::*tprA2* (black bars) individually. The data is represented as ratios relative to the geometric mean of housekeeping genes *gyrB* and *metG* (Y-axis). Target genes are indicated on the X-axis. Error bars represent standard deviations. Statistical significance was determined by Student’s *t*-test and was calculated with reference to WT in each set of test gene; ‘*’, *P*-value = <0.05; ‘**’, *P*-value<0.01.

We investigated the regulation of the TprA2/PhrA2 system *in vivo* to verify whether our *in vitro* finding were relevant to the *in vivo* environment. We analyzed WT, Δ*tprA2*, and Δ*tprA2*::*tprA2*. Three chinchillas were independently inoculated with each strain, middle ear effusions were extracted 48 hours post-inoculation, and bacterial mRNA for *tprA2*, *ABCATPase*, *lcpA* and *lcpM* was quantified using nanostring technology. As observed *in vitro*, deletion of *tprA2* led to increase expression of *ABCATPase* (on the same transcript as *phrA2*) and *lcpM* (Fig 7). *LcpA* values were also higher in this mutant, but display elevated inter-animal variability such that the change was not statistically significant. The modest fold increase is consistent with our observation that the TprA2-regulon in the WT is highly expressed *in vivo*, such that complete removal of the negative regulator has a moderate effect. In contrast, overexpression of *tprA2* in the complement strain led to a decrease in the levels of *ABCATPase* and *lcpA*. Together, these findings suggest TprA2 is negative regulator of its neighboring genes *in vivo*.

### TprA2 promotes commensalism over tissue dissemination

To assess the *in vivo* role of the QS-Lcp region we made use of two pneumococcal infection models. To study colonization of the nasopharynx and spread to the lungs we utilized a murine model where animals are inoculated intranasally and disease progresses causing pneumonia or sepsis or both [36,37]. To study middle ear disease we utilized the chinchilla otitis media model.

**Fig 8:**
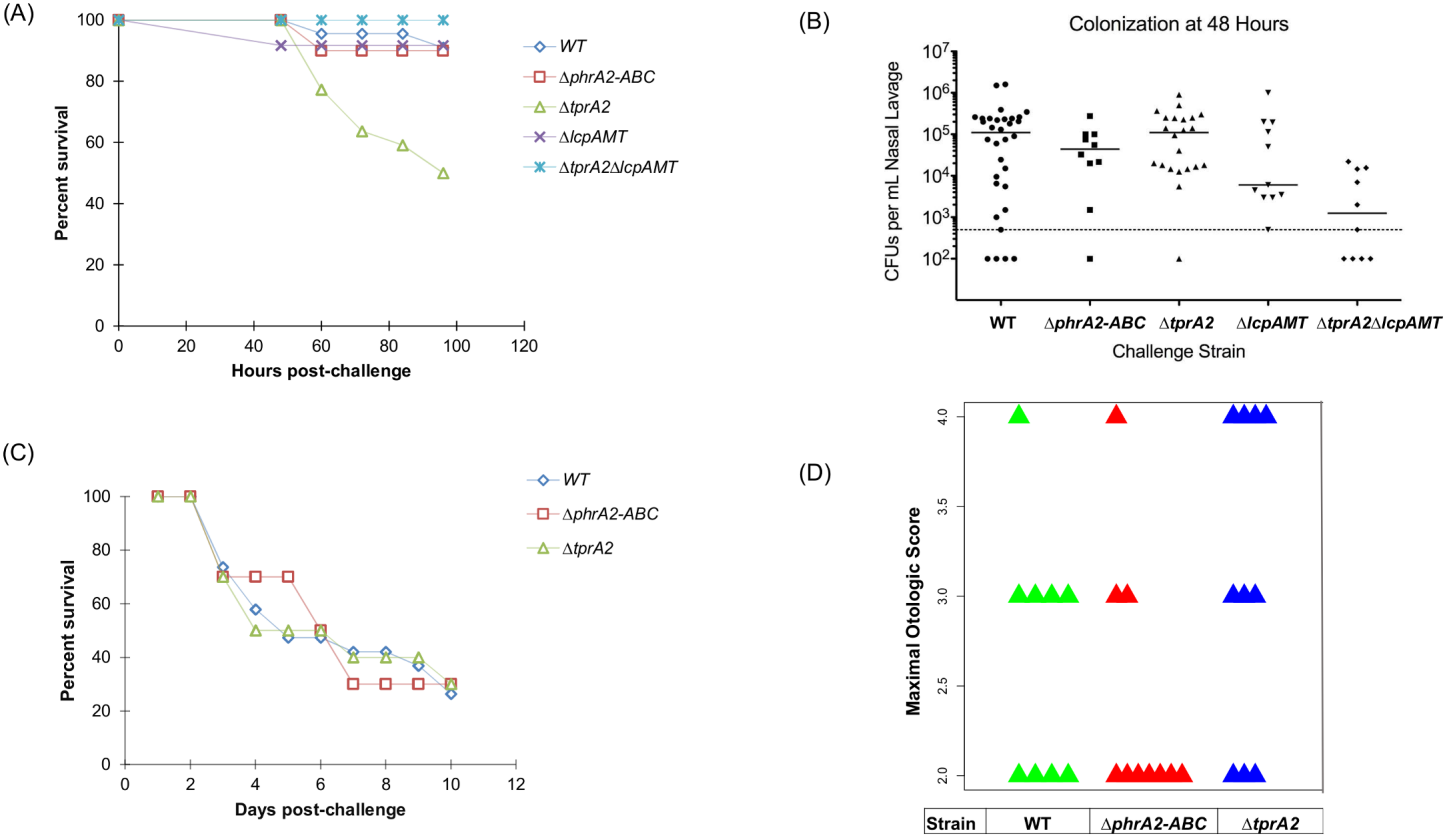
*In vivo* effects of TprA2/PhrA2 system. **(A,B)** Analysis of PMEN1 strain SV36 WT and isogenic mutants **Δ***tprA2*; **Δ***phrA2-ABC*; **Δ***lcpAMT;* and Δ*tprA2*Δ*lcpAMT* in the murine model with intranasal inoculations. (A) Percentage survival of mice after intranasal inoculation. Cohorts of at least ten mice were assessed for the duration of four days. Statistical significance relative to WT was calculated using Mann-Whitney U test; ‘*’, *P*-value<0.05. (B) Bacterial counts from nasal lavages of mice 48h post-inoculation. (C,D) Analysis of PMEN1 strain (4595-T23) WT and isogenic mutants Δ*tprA2* and Δ*phrA2-ABC* in the chinchilla model of otitis media. (**C)** Percentage survival of chinchillas after transbullar inoculation. Cohorts of at least ten chinchillas were assessed for the duration of ten days. **(D)** Scatter plots illustrate the maximal otologic score for animals infected with WT (green), Δ*phrA2-ABC* (red) or Δ*tprA2* (blue). Each triangle represents one animal. Otologic disease ranged from no disease to a ruptured tympanic membrane, where a score of ‘1’ is given for animals with mild or no disease, ‘2’ with moderate disease, ‘3’ with frank purulence, and “4” with tympanic membrane rupture.

The murine model revealed that TprA2 protects against lung disease. We did not observe infection in mice inoculated with PN4595-T23 strains, thus we generated the parallel mutants in another naturally occurring PMEN1 strain with a type 3 capsule (SV36). Cohorts of ten BALB/c mice were infected with SV36, SV36Δ*tprA2* or SV36Δ*phrA2*-*ABC* and observed over 4 days. The bacterial titers in the nasal lavages were similar for all three strains when tested at 48 hours post-inoculation (Fig 8B). Notably, SV36Δ*tprA2* displayed a statistically significant increase in mortality (Fig 8A).

TprA2 is a negative regulator of *lcpAMT* (Fig 5). To test whether overexpression of *lcpAMT* in the SV36Δ*tprA2* was associated with the increase virulence of this strain, we tested a double mutant with deletions in *tprA2* and *lcpAMT* and observed that it restored the wild-type phenotype. These results strongly suggest that LcpA is a virulence determinant, and that TprA2 can modulate virulence by controlling levels of *lcpAMT*.

Finally, to study middle ear disease, bacteria were inoculated directly into the middle ear of chinchillas. The overall mortality was the same for all three strains, perhaps reflecting differences in peripheral disease progression from the chinchilla middle ear versus the murine nasopharynx (Fig 8C). Further, we observed a trend toward increased middle ear disease in the Δ*tprA2* (Fig 8D), and the Δ*tprA2* displayed the highest lung dissemination (S3 Table), consistent with our finding that *lcpAMT* plays a role in virulence. In conclusion, our findings suggest that TprA2 controls *lcpA* expression and in doing so can promote commensalism over dissemination.

### PMEN1 codes for two related regulator/peptide systems

TprA2 shares moderate homology to TprA, another streptococcal transcription factor that belongs to the recently characterized TprA/PhrA system, where TprA inhibits expression of PhrA and downstream lantibiotic genes [24]. Unlike *tprA2*, which occurs rarely outside the PMEN1 lineage, *tprA* has a wide distribution in pneumococci. Using a set of highly curated WGSs, with representatives of the major lineages of *S*. *pneumoniae*, we found that *tprA* was present in over 90% of the isolates in our set (Fig 2, all *tprA* genes displayed > = 86% similarity). The prominent exception is a set of strains in a basal pneumococcal branch associated with unencapsulated strains and conjunctivitis infections [38,39] (Fig 2). Hoover and colleagues first characterized the TprA/PhrA system, and also reported a wide distribution (approximately 60%) in pneumococcal strains [24].

**Fig 9:**
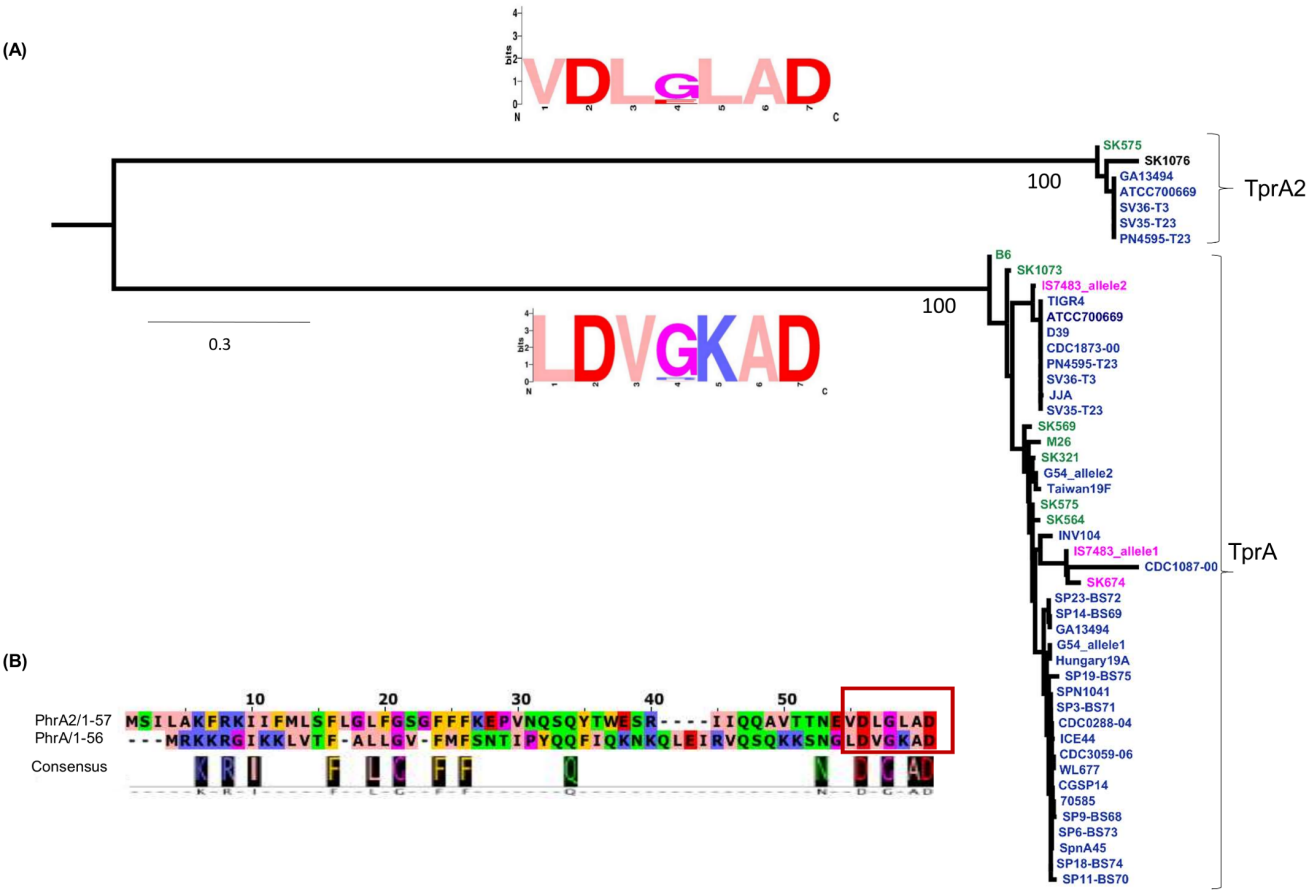
Phylogenetic analysis of separation between TprA2 and TprA systems. **(A)** Gene tree generated from the coding sequences for *tprA* and *tprA2* using maximum likelihood. Each branch displays a sequence logo, derived from the predicted C-terminal heptapeptide of PhrA2 (top) and PhrA (bottom). In the logo, amino acids are represented in one letter abbreviation where their height within the stack represents its relative frequency at a given position, in zappo color-coding scheme: blue/positive; red/negative; salmon/hydrophobic; orange/aromatics; purple/glycine or proline; green/hydrophilic. **(B)** Alignment of predicted coding sequence of PhrA and PhrA2 in PMEN1 strain PN4595-T23. Representation showing alignment (top) and consensus (bottom). Seven amino acids of the C-termini are highlighted in the red box indicating the sequence of synthetic peptides used in this study.

PMEN1 strains are notable in that they code for both the TprA2/PhrA2 and TprA/PhrA QS systems. In the PMEN1 strain PN4595-T23, the TprA and TprA2 protein sequences share approximately 60% identity. We searched the genomes of 55 streptococcal strains, identified 48 sequences to construct a phylogenetic tree of these regulators using maximum likelihood, and found that the *tprA2* and *tprA* homologues are separated into two distinct branches (Fig 9A). Their cognate peptides in PMEN1, PhrA2 and PhrA share only 28% identity over the full length, but display very high similarity at their C-termini. To analyze the extent of conservation of the C-terminal residues, we generated a consensus logo from the six PhrA2 sequences and the thirty-six PhrA sequences. The C-terminal residues are either identical or share similar charge in 6/7 residues; but can be distinguished by position -3 that codes for a conserved leucine in PhrA2 and a lysine in PhrA (Fig 9A and 9B). The sequence separation between the QS components suggests that the *tprA2/phrA2* genes did not originate from a recent duplication within PMEN1, and is consistent with acquisition of TprA2/PhrA2 by horizontal gene transfer.

### Interaction of TprA2/PhrA2 QS system with the TprA/PhrA QS system

**Fig 10:**
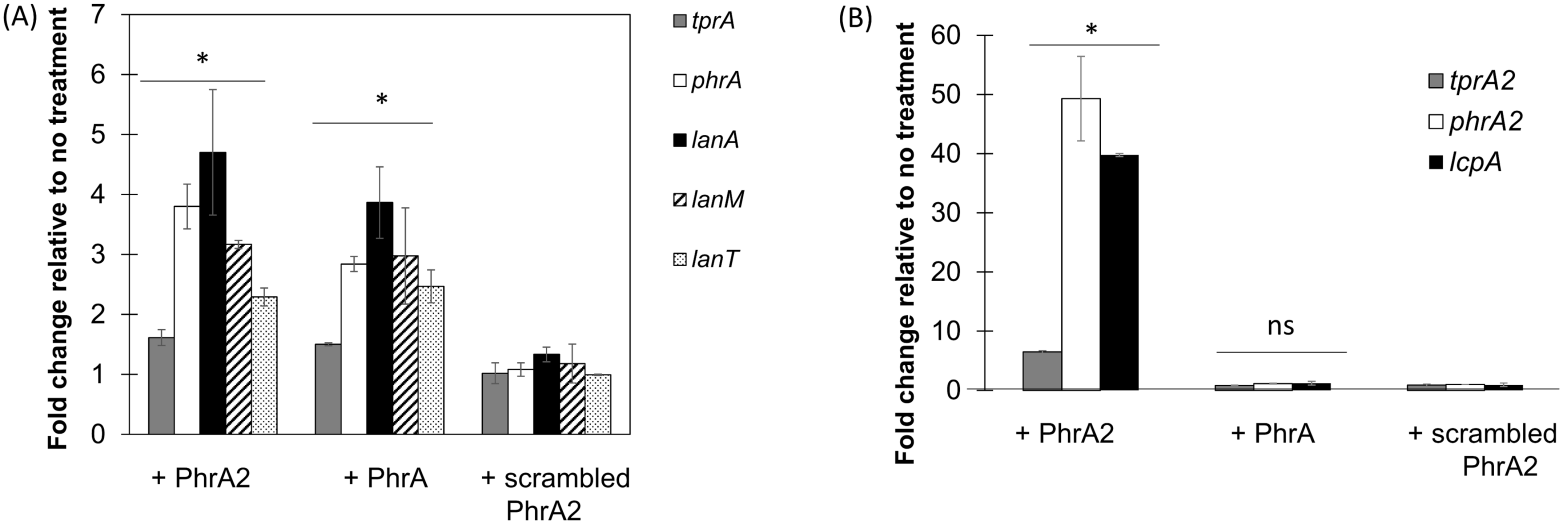
PhrA2 influences the gene expression levels of the TprA/PhrA system. qRT-PCR measurements of gene expression for target genes performed in strain PN4595-T23. Data was normalized to levels of 16S rRNA. The X-axis denotes test genes of TprA/PhrA system and treatment conditions. The Y-axis reflects the fold change in the treatment group relative to the no treatment control. Treatments correspond to: (i) PhrA2 C-terminal heptapeptide (VDLGLAD); (ii) PhrA C-terminal heptapeptide (LDVGKAD); or (iii) scrambled peptide (DAGVLDL). Error bars represent standard deviations for biological replicates (n = 3). (A) Target genes correspond to *tprA* regulator (gray bar), its cognate *phrA* peptide (white bar), and lantibiotic genes in the TprA regulon (*lanA*/dark bar; *lanM* stripped bar; and *lanT*/dotted bar). * Statistically significant difference in gene expression compared to scrambled peptide (*P*-value<0.05). (B) Target genes correspond to *tprA2* regulator (gray bar), its cognate *phrA2* peptide (white bar), and *lcpA* /dark bar. * Statistically significant difference in gene expression compared to scrambled peptide (*P*-value<0.01, ns = not significant).

The co-occurrence of both QS systems in the PMEN1 strains led us to investigate whether PhrA2 and PhrA peptides can exert regulatory effects on their non-cognate QS systems, TprA/PhrA and TprA2/PhrA2 respectively. To test this, we measured how the addition of synthetic peptides to the extracellular milieu affects gene expression of the non-cognate regulon. Addition of synthetic PhrA2 (VDLGLAD), but not the scrambled peptide, induced gene expression of the TprA regulon (*tprA*, *phrA*, and the TprA-associated *lanA*, *lanM*, and *lanT*) at levels similar to those induced by cognate PhrA (LDVGKAD) itself (Fig 10A). In contrast, neither the addition of synthetic PhrA nor the addition of the scrambled peptide had any effect on expression of the *tprA2*, *phrA2*, or *lcpA* genes in the TprA2/PhrA2 regulon (Fig 10B). These findings suggest that PhrA2 regulates gene expression of the TprA regulon, and PhrA has no effect on the TprA2 regulon.

### PhrA2 regulates the TprA/PhrA system in non-PMEN1 strains

The unidirectional influence of PhrA2 gene expression upon TprA/PhrA led us to investigate whether the PMEN1 peptide could influence gene expression in non-PMEN1 cells. We used strain D39 as a representative of the non-PMEN1 strains since TprA/PhrA system has been previously described in D39. Hoover *et al*. have demonstrated that *phrA* is under catabolite repression.

The gene encoding *phrA* is expressed in galactose and repressed in glucose, and the *phrA* promoter region contains a *cre* (catabolite response element) site for CcpA catabolite repression [24,40]. In contrast, we have not identified a *cre* site in the *phrA2* promoter region. Therefore, to maximally discern the input through PhrA2 in our experiment, we used a D39-derived strain with a deletion of *phrA* and grew it in chemically-defined medium with galactose as the sole sugar.

**Fig 11:**
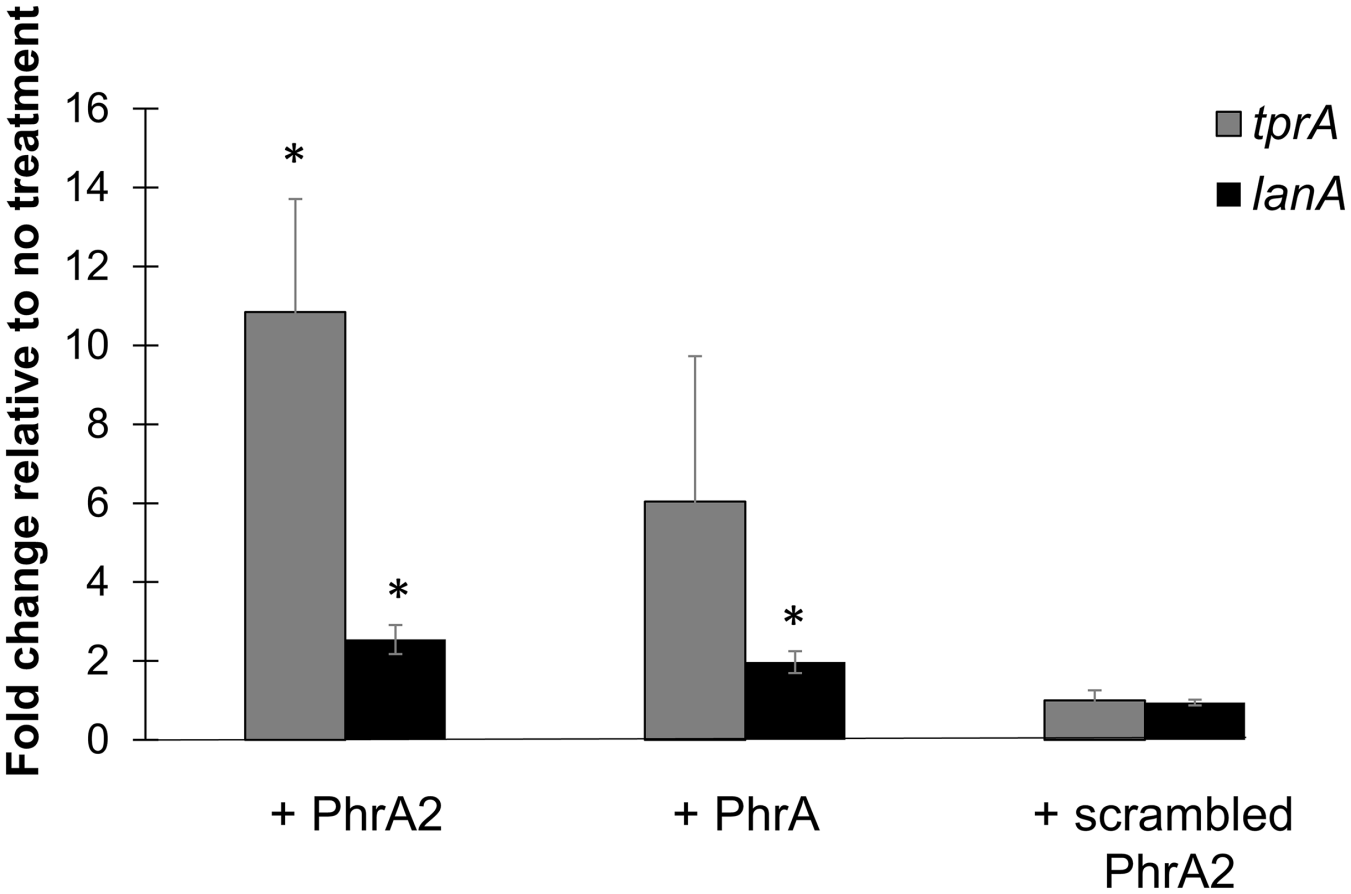
PhrA2 influences the gene expression levels of the TprA/PhrA system in non-PMEN1 strain D39. qRT-PCR measurements of gene expression for target genes performed in strain *D39ΔphrA* upon treatments indicated on the X-axis. Data was normalized to levels of 16S rRNA. The Y-axis reflects the fold change in the treatment group relative to the no treatment control. Treatments correspond to: (i) PhrA2 C-terminal heptapeptide (VDLGLAD); (ii) PhrA C-terminal heptapeptide (LDVGKAD); or (iii) scrambled peptide (DAGVLDL). Target genes correspond to *tprA* regulator (gray bar), and its associated *lanA* gene (black bar). Error bars represent standard deviations for biological replicates (n = 3), * Statistically significant difference in gene expression compared to scrambled peptide (*P*-value<0.01).

We found that exogenous PhrA2 interacts with the TprA regulon in non-PMEN1 strains. Specifically, D39Δ*phrA* cultures were exposed to treatments with synthetic PhrA2, PhrA, and scrambled peptides for an hour and gene expression of *tprA* and *lanA* was measured relative to no treatment. Treatment with PhrA2 significantly induced expression of *tprA* and *lanA* by 11-fold and 2-fold, respectively (Fig 11). Treatment with scrambled peptide showed no induction of gene expression in *D39ΔphrA*. The extent of *lanA* induction by PhrA is lower in the D39Δ*phrA* strain than in experiments with the WT strain (Fig 10A), we presume this difference is due to the absence of *phrA*-autoinduction in the mutant strain. These findings suggests that PhrA2 can be internalized by strains outside the PMEN1 lineage and induce changes in their gene expression.

## Discussion

Our findings demonstrate that acquisition of the TprA2/PhrA2 QS system by horizontal gene transfer into the PMEN1 lineage has endowed these strains with a virulence determinant and a mechanism to regulate its expression and thereby control disease. PMEN1 (ST81) lineage is postulated to have evolved from an ancestor in 1967, and by the end of 1990s it represented an estimated 40% of penicillin resistant strains in US [14,41]. These strains display very high rates of carriage [2,3,41,42]. PMEN1 also displays very high rates of disease [2,3,43]. Is the prevalence of PMEN1 in invasive disease a function of its carriage rates or does it reflect a propensity to cause disease? Multiple studies have shown that sequence types vary regarding their propensity to cause disease [44–47] and Sjostrom *et al*. show that PMEN1 displays a low propensity to cause invasive disease [47]. Thus, high rates of PMEN1 invasive disease in the population likely reflect high carriage rates, and not heightened virulence potential. In this context, it is possible that acquisition of the TprA2/PhrA2 by PMEN1 strains contributes to its low proclivity to cause invasive disease.

**Fig 12:**
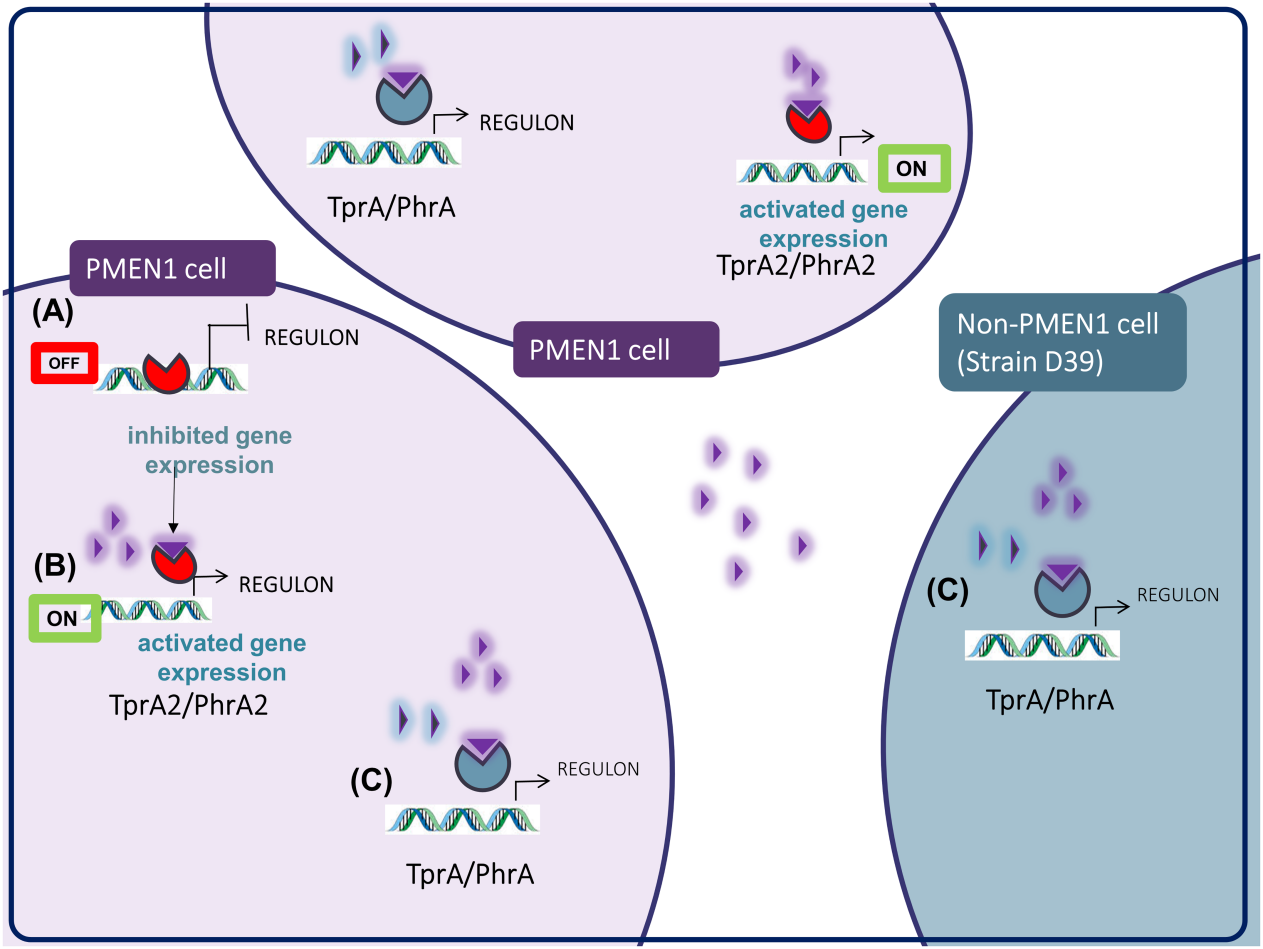
Model for regulation of gene expression by TprA2-PhrA2. (A) In the OFF state, TprA2 inhibits gene expression. (B) In the ON state, PhrA2 releases TprA2-mediated gene inhibition. This effect of PhrA2 is observed from synthetic peptide added to the extracellular milieu and cell-free supernatant, suggesting that PhrA2 is exported, activated and re-imported before it modulates TprA2 activity, in both the producer PMEN1 cells and surrounding PMEN1 population. (C) PhrA2 secreted by PMEN1 cells activates gene expression of *tprA* and associated *lanA*, in both PMEN1 and non-PMEN1 cells. Red circular shape/TprA2, purple triangle/PhrA2, blue circular shape/TprA; blue triangle/PhrA.

TprA2/PhrA2 may provide PMEN1 strains with the means to manipulate gene expression in neighboring strains from other lineages in multi-strain infections. We show that synthetic C-terminal PhrA2 can stimulate expression of the TprA/PhrA system as well as its associated lantibiotic biosynthesis cluster in distantly related strain D39 (Figs 11 and 12). We have observed that the expression of PMEN1-*phrA2* is six fold that of D39-*phrA* in rich media, thus exemplifying a condition where PMEN1-*phrA2* expression is high when D39-*phrA* is low (S3 Fig). We are currently investigating this interaction in physiologically relevant conditions. The activation of *phrA* in response to galactose has led to the conclusion that TprA/PhrA may promote colonization in the nasopharynx where free sugars are rare and pneumococci survive by breaking down host mucins to free complex sugars, most prominently galactose [24]. However, experiments with TprA/PhrA in the murine model demonstrate that this system is a virulence determinant in multiple models of pneumococcal disease (personal communication, Motib and Yesilkaya), in this manner, PhrA2 may trigger a virulence regulon in neighboring strains. We propose that PhrA2 signaling across systems is physiologically relevant in multi-strain infections.

We conclude that PhrA2 peptide is secreted by PMEN1-cells, since cell-free culture supernatants reiterate the function of extracellular addition of synthetic PhrA2. We predict that export occurs via the Sec secretion system, consistent with other peptides from the PlcR family of regulator-peptide pairs [48–50]. Import must occur via a relatively widespread transporter, given that PhrA2 can influence D39 gene expression. Further, the high sequence similarity between the functional C-termini of PhrA and PhrA2 suggests common import machinery. The oligopeptide permease *amiACDEF* has been shown to be required for import of processed PhrA, and its homologues are required for import of PlcR-associated peptides in other species [48–50]. Thus, *amiACDEF* is a high value candidate for a PhrA2 importer.

Sequence comparisons suggest that LcpA is a bacteriocin, however its function remains unknown. We propose that its effect on virulence is not the result of bacteriocidal activity given that mouse experiments where performed with single strains. However, we cannot exclude the possibility that an interaction between LcpA and the natural microbiome of the mouse influences the outcome of the infection. The function of LcpA is under investigation.

We have identified and characterized a new quorum sensing system from the emerging RRNPP family. TprA2/PhrA2 consists of a negative regulator of a lanthionine containing peptide and a cognate activating peptide. Our findings suggest that this system has provided PMEN1 with the ability to control LcpA virulence and perhaps influence its propensity to cause invasive disease. Finally, to our knowledge this is the first example of a gene transfer event that has integrated with an ancestral regulatory networks to control inter-strain gene regulation.

## Materials and methods

### Ethics statement

Laboratory animals were maintained in accordance with the applicable portions of the Animal Welfare Act and the guidelines prescribed in the DHHS publication, Guide for the Care and Use of Laboratory Animals. The Office of Laboratory Animal Welfare (OLAW) Assurance of Compliance number is A3693-01. All chinchilla experiments were conducted with the approval of the Allegheny-Singer Research Institute (ASRI) Institutional Animal Care and Use Committee (IACUC) A3693-01/1000. Research grade young adult chinchillas (*Chinchilla lanigera*) weighing 400–600 grams were acquired from R and R Chinchilla Inc., Ohio. Animals were maintained in BSL2 facilities and all experiments were done while chinchillas were under subcutaneously injected ketamine-xylazine anaesthesia (1.7mg/kg animal weight for each). For virulence studies, chinchillas (a minimum of 10 in each cohort) were infected with 100 CFUs/ear by transbullar inoculation within each middle ear. During the course of the experiment (10 days), animals with severe acute infection perished; animals showing prolonged signs of discomfort were administered with pain relief (Rimadyl, 0.1ml of 50mg/mL)). Animals with severe signs of pain and illness were euthanized by administering an intra-cardiac injection of 1mL potassium chloride after regular sedation. All experiments involving mice were performed with prior approval of and in accordance with guidelines of the St. Jude Institutional Animal Care and Use Committee. The St Jude laboratory animal facilities have been fully accredited by the American Association for Accreditation of Laboratory Animal Care. All mice were maintained in BSL2 facilities and all experiments were done while the mice were under inhaled isoflurane (2.5%) anesthesia. Mice were monitored daily for signs of infection. This work was approved under the IACUC protocol number 538-100013-04/12 R1. Mice were monitored for disease progression and euthanized via CO_2_ asphyxiation.

### Comparative genomics

We performed a comparative genomic analysis of PMEN1 and non-PMEN1 strains to identify genes unique to the PMEN1 lineage [27]. To this end, we used a set of 60 curated pneumococcal whole-genome sequences (WGS), including four from the PMEN1 lineage (S1 Table). The set of 60 genomes includes the 44 genomes used for the first large-scale pneumococcal pangenome study [8], additional genomes from PCV-7 immunized children [51], as well as genomes from non-encapsulated strains [52]. Together these strains reflect a large variety of multilocus sequence types (MLSTs) and serotypes, as well as strains isolated from different disease states and geographic locations.

To determine the distribution of *tprA2* across pneumococcal strains we searched for this gene in the genome sequence of 215 PMEN1 isolates [13]. A few genomes displayed disruption in the *tprA2* locus, so the sequences were confirmed by PCR. Primers to *tprA2* and *gapdh* (positive control) were used to amplify these respective genes from genomic DNA. The genomes from strains 111 (ERS004810), 11933 (ERS005313) and HKP38 (ERS004775) display substantial differences in the locus encoding TprA2/PhrA2.

To search for *cre* sites we inspected the 190 basepairs upstream of *phrA2* and before the start of *tprA2*. We searched for the *cre* site motif from *L*. *lactis* (WGWAARCGYTWWMA), and allowed for up to three discrepancies as has been observed in a subset of *S*. *pneumoniae cre* [40,53].

### Bacterial strains and growth conditions

Wild-type *S*. *pneumoniae* strains PN4595-T23 (GenBank ABXO01) and SV36 (GenBank ADNO01), graciously provided by Drs. Alexander Tomasz and Herminia deLancastre, were used as PMEN1 representatives [31]. Strains 111 (ERS004810), 11933 (ERS005313) and HKP38 (ERS004775) were shared by Drs. Julian Parkhill and Stephen Bentley, and originally obtained from Drs. Lesley McGee, Mark can der Linden, So Hyun Kim and Jae Hoon Song.

For growth on solid media, *S*. *pneumoniae* (PN4595-T23) and isogenic mutants were streaked on TSA II plates with 5% sheep blood (BD BBL, New Jersey, USA). For growth in liquid culture, colonies from a frozen stock were grown overnight on TSA plates, inoculated into Columbia broth (Remel Microbiology Products, Thermo Fisher Scientific, USA), and incubated at 37°C and 5% CO2 without shaking. Columbia broth contains 10mM glucose. Experiments in chemically defined media (CDM) were performed utilizing previously published recipe [40], and galactose was used at a final concentration of 55mM. Growth in CDM was initiated by growing a pre-culture for 9 hours and back dilution to OD_600_ 0.1 to initiate a culture.

### Generation of deletion mutants and complement strains

All deletion mutant strains were generated by site-directed homologous recombination where the target region was replaced with the spectinomycin-resistance gene (*aadR*) or kanamycin-resistance gene, as previously described [27] [54]. Briefly, ~2kb of flanking region upstream and downstream of the deletion target were amplified from the parental strain by PCR using Q5 2x Master Mix (New England Biolabs, USA) generating flanking regions, and the spectinomycin resistant gene was amplified from the plasmid pR412 (provided by Dr. Donald Morrison). Assembly of the transforming cassette was achieved either by sticky-end ligation of restriction enzyme-cut PCR products or by Gibson Assembly using NEBuilder HiFi DNA Assembly Cloning Kit. The resulting construct was transformed into PN4595-T23 and confirmed using PCR and DNA sequencing.

Complement strains were made by generating a cassette where ~100bp of the 5’UTR and the CDS of the gene to be complemented were fused at the 3’ end of an antibiotic selection cassette lacking a transcription terminator. This cassette was introduced in the genome of the strain at one of the two regions: the intergenic region between the orthologues of spr_0515 and spr_0516, an inert genomic region that has been successfully employed in other constructs in the lab, or the *bga* region a commonly employed site for complementation [55]. After subsequent transformation, qRT-PCR (LightCycler480, Roche Life Sciences, USA) was done to verify the levels of expression of the complemented gene. Primers used to generate the constructs are listed in S4 Table.

### Bacterial transformations

For all bacterial transformations, about 1μg of transforming DNA was added to the growing culture of a target strain at OD_600_ of 0.05, supplemented with 125μg/mL of CSP2 (sequence: EMRISRIILDFLFLRKK; purchased from GenScript, NJ, USA), and incubated at 37°C. After 4 hours, the treated cultures were plated on Columbia agar containing the appropriate concentration of antibiotic for selection; spectinomycin, 100μg/mL; erythromycin 2μg/mL, kanamycin 150μg/mL). Resistant colonies were cultured in media, the region of interest was amplified by PCR and the amplimer was submitted for Sanger sequencing (Genewiz, Inc., USA) to verify the sequence of the mutants. The strains generated in this study are listed in Table 2.

**Table 2:** Strains used in this study.

Strain	Description	Source
PN4595-T23	PMEN1 strain isolated in Lisbon, Portugal in 1996	Drs. A.Tomasz and H. deLencastre
Δ*tprA*2	PN4595-T23; CGSSp4595_1262:Spec^R^	This study
Δ*tprA2*::*tprA2*	*tprA2* gene inserted in the intergenic region of equivalent genes spr_0515 and spr_0516 with 90 bp of native promoter downstream of constitutively expressed erythromycin resistant gene (*ermB*)	This study
Δ*phrA2-ABC*	PN4595-T23; CGSSp4595_1258-CGSSp4595_1261:Spec^R^	This study
		This study
SV36	PMEN1 strain isolated in New York in 1996	Drs. A.Tomasz and H. deLencastre
SV36*ΔtprA2*	SV36; CGSSp4595_1262:Spec^R^	This study
SV36*ΔphrA2-ABC*	*phrA2* and downstream ABC transporters (CGSp4595_1261, CGSSpSV36_1147, CGSSpSV36_1146, CGSSpSV36_1145) replaced with spectinomycin resistant gene in SV36	This study
SV36Δ*tprA2*Δ*lcpAMT*	SV36;CGSSp4595_1262:Spec^R^;CGSSp4595_1257,1256:Kan^R^	This study
SV36Δ*lcpAMT*	SV36;CGSSp4595_1257,1256:Kan^R^	This study
D39Δ*phrA*	D39; SPD_1746:Spec^R^	Dr. H. Yesilkaya

### Treatment with synthetic peptides

Bacterial cultures were treated with synthetic peptides corresponding to the following sequences: 1) C-terminal PhrA2 heptamer (VDLGLAD); 2) C-terminal PhrA heptamer (LDVGKAD); and 3) scrambled peptide comprised of the same residues as the PhrA2 heptamer (DAGVLDL). These were custom ordered from GenScript, (NJ, USA) at 99.7% purity. 1μM peptide was added in the mid-log phase (OD_600_ of 0.5), cultures were incubated at 37°C, 5% CO_2_ for 1 hour, after which RNA later (Ambion^®^, Thermo Fisher Scientific, USA) was added to the cultures to preserve RNA and subsequent RNA extraction and qRT-PCR were performed.

For experiments where different peptides were compared in parallel, the original culture was distributed into separate tubes, and each one was treated with the relevant peptide, in addition to a no-peptide control. Using a single parent culture for different peptide additions ensured minimal variation when comparing treatments.

### Treatment with cell-free supernatant

To determine whether secreted peptides can stimulate gene expression in a recipient wild-type culture, recipient cultures and supernatant donor cultures were grown alongside to selected OD_600_. To prepare cell-free supernatant, bacterial cells were pelleted and the supernatants were filtered (pore size 0.2 microns). At the desired OD_600_, the wild-type recipient culture was distributed into separate tubes, cultures were centrifuged at 4000g for 7 minutes, and resuspended in the same volume of cell-free supernatant or media control. At 1 hour post-treatment, RNA later (Ambion^®^, Thermo Fisher Scientific, USA) was added to each culture, and samples were prepared for RNA extraction and qRT-PCR.

### Preparation of cell lysates and RNA collection, extraction, and quality assessment

For experiments on *in vitro* transcriptional analysis, samples were collected for RNA extraction at an OD_600_ of 0.5 unless otherwise stated and preparation of RNA was performed as previously described in [34]. For RNA extraction from *in vivo* experiments, chinchillas were euthanized 48h post-inoculation of PN4595-T23, and a small opening was generated through the bulla to access the middle ear cavity. Effusions were siphoned out from the middle ear and flash frozen in liquid nitrogen to preserve the bacterial RNA. For bacterial cell lyses, the sample were re-suspended in an enzyme cocktail (2mg/mL proteinase K, 10mg/mL lysozyme and 20μg/mL mutanolysin), and submitted to bead beating with glass beads, acid-washed 425–600μm (Sigma) and 0.5mm Disruption Beads made by Zirconia/Silica in FastPrep-24 Instrument (MP Biomedicals, USA). These cell lysates were frozen for microarray, qRT-PCR or nanoString analyses. The RNA concentration was measured by NanoDrop 2000c spectrophotometer (Thermo Fisher Scientific, USA) and its integrity was confirmed on gel electrophoresis.

### Microarray analyses of gene expression levels

We utilized the Pneumococcal Supragenome Hybridization Array (SpSGH) to compare gene expression between the wild-type PN4595-T23 strain and the *ΔtprA2* [34]. The array provides coverage for ~85% of the PMEN1 open reading frames. Strains were grown to mid-log cultures (OD_600_ 0.5) in Columbia broth (note, that glucose in the media will inhibit genes under catabolic repression). RNA extraction, cDNA preparation and cDNA labeling were performed as previously described [34]. Cyber T was used for data analysis [56,57]. Genes with at least a 10-fold difference between strains and Bayesian *P* values < 0.05, Benjamini-Hochberg FDR < 10%, and Bonferroni-corrected *P* value < 0.05 are displayed in Table 1. The complete dataset is deposited in GEO web storage (under submission).

### qRT-PCR analyses of gene expression levels

High quality RNA (DNA free and A_260/280_ ~ 2.1) was used as template for the synthesis of first strand of cDNA using SuperScript VILO synthesis kit (Invitrogen). After first strand cDNA synthesis, the product was directly used for qRT-PCR using LighCycer480 Master Mix SYBRGreen in a LightCycler480 Instrument (Roche Life Sciences, USA). For normalization, we used 16S rRNA, as well as *gyrB* (DNA gyrase subunit B) and/or *gapdh* (glyceraldehyde-3-phosphate dehydrogenase). The raw data was converted using LC480 Conversion: conversion of raw LC480 data” software (available at http://www.hartfaalcentrum.nl/index.php?main=files&sub=0) and LinregPCR for expression data analysis [58,59], where the output expression data is displayed in arbitrary fluorescence units (N_0_) that represent the starting RNA amount for the test gene in that sample. Statistical significance was determined by performing Student *t*-test (unpaired samples, one tailed), using GraphPad Prism 6 tool.

### NanoString technology for *in vivo* gene expression

nCounter Analysis System from nanoString technology provides a highly sensitive platform to measure gene expression of a pathogen during host infection [60]. The fully-automated, barcode technology directly detects mRNA transcripts, thereby eliminating the amplification and enzymatic steps of DNase treatment and cDNA synthesis. The probes used in our study were custom designed by nanoString Technologies and included housekeeping genes *gyrB* and *metG* as normalization controls (S4 Table). Nanostring probes for long coding sequences were generated and probes for *phrA2* could not be manufactured. 5μL of extracted RNA samples, collected directly from processing of middle ear effusions with the RNeasy Mini Kit, were hybridized onto the nCounter chip following manufacture’s instruction. RNA concentration ranged from 80–200 ng/μl for *in vivo* samples, and 50ng total nucleic acid for planktonic samples. Manufacturer’s software, nSolver, was used for quality assessment of the raw data and normalization. The data was normalized across samples against the geometric mean of the housekeeping genes, *gyrB* and *metG* [40,61]. 16srRNA and *gapdh* were not used as *in vivo* controls, given the very high abundance of 16SrRNA that overwhelms the nanoString signal, and the evidence of a role for GAPDH during infection that may led higher expression *in vivo* [62]. Finally, the *in vitro* and *in vivo* levels were compared using Student’s *t*-test in the GraphPad Prism 6 tool.

### Virulence studies in the chinchilla OM model

All chinchilla experiments were conducted with the approval of the Allegheny-Singer Research Institute (ASRI) Institutional Animal Care and Use Committee (IACUC) A3693-01/1000. Research grade young adult chinchillas (*Chinchilla lanigera*) weighing 400–600 grams were acquired from R and R Chinchilla Inc., Ohio. Animals were maintained in BSL2 facilities and all experiments were done while chinchillas were under subcutaneously injected ketamine-xylazine anaesthesia (1.7mg/kg animal weight for each). For virulence studies, chinchillas (a minimum of 10 in each cohort) were infected with 100 CFUs/ear by transbullar inoculation within each middle ear. During the course of the experiment (10 days), animals with severe acute infection perished; animals showing prolonged signs of discomfort were administered with pain relief (Rimadyl, 0.1ml of 50mg/mL)). Animals with severe signs of pain and illness were euthanized by administering an intra-cardiac injection of 1mL potassium chloride after regular sedation. We evaluated mortality, time to death, and spread of bacteria to the brain and the lungs. Tissue dissemination was tested by plating homogenized tissue on TSA plates with 5% sheep blood to establish pneumococcal presence. Additionally, we assessed local diseases using visual otoscopic inspection (VetDock, USA). Otologic disease ranged from no disease to a ruptured tympanic membrane, where a score of ‘1’ is given for animals with mild or no disease, ‘2’ with moderate disease (where pus and air are present), ‘3’ with frank purulence, and ‘4’ with tympanic membrane rupture [7,63].

### Virulence studies in the murine lung model

All experiments involving mice were performed with prior approval of and in accordance with guidelines of the St. Jude Institutional Animal Care and Use Committee. The St Jude laboratory animal facilities have been fully accredited by the American Association for Accreditation of Laboratory Animal Care. Laboratory animals were maintained in accordance with the applicable portions of the Animal Welfare Act and the guidelines prescribed in the DHHS publication, Guide for the Care and Use of Laboratory Animals. All mice were maintained in BSL2 facilities and all experiments were done while the mice were under inhaled isoflurane (2.5%) anesthesia. Mice were monitored daily for signs of infection. This work was approved under the IACUC protocol number 538-100013-04/12 R1. For bacterial burden and survival studies, strains were grown in C+Y media to an OD_620_ of 0.4 and diluted according to a previously determined standard curve. Bacteria were enumerated to assure that the proper amount of bacteria was used in infection. Bacteria were introduced into 7-week-old female BALB/c mice (Jackson Laboratory) via intranasal administration of 5 x10^4^ CFU of bacteria in PBS (100 μL). Mice were monitored for disease progression and euthanized via CO_2_ asphyxiation. Blood for titer determination was collected via tail snip at 24 and 48 hours post-infection and subsequent serial dilution and plating. Bacteria colonizing the nasopharynx were collected by insertion and removal of PBS (20 μL) into the nasal cavity. One cohort was used for Δ*phrA2-ABC*, Δ*lcpAMT*, and Δ*tprA2*Δ*lcpAMT*, while two cohorts were used for WT and Δ*tprA2* (Fig 8A and 8B). Survival data were analyzed using the Mann-Whitney U test in Prism 6. Bacterial titers were compared using nonparametric Mann-Whitney U t test in Prism 6.

### Generation of phylogenetic trees and their analyses

#### Generation of streptococcal species tree

Fifty-five streptococcal strains were selected for phylogenetic analysis (S1 Table, labeled “Distribution within Streptococcus sp.”). The 33 pneumococcal strain were selected to capture the major sequence clusters within this species, including 4 PMEN1 genomes given the focus of this manuscript on this lineage. The *S*. *mitis* and *S*. *pseudopneumoniae* strains represented the available genomes for these species at the time this study was initiated. The *S*. *tigurinus* were selected as a potential novel species related to *S*. *mitis* [64]. According to our analysis, the *S*. *tigurinus* genomes and a subset of the *S*. *mitis* genomes cluster with *S*. *oralis*. The whole genome sequence (WGS) for all 55 strains were aligned using MAUVE [65,66] and the core region corresponding to 995531 total sites and 352,371 informative sites, was extracted from the Mauve output files. Alignment of the core region was performed using MAFFT (FFT-NS-2) [67] and model selection was performed using MODELTEST [68]. The phylogenetic tree was built with PhyML 3.0 [69], model GTR+I(0.63) using maximum likelihood and 100 bootstrap replicates.

#### Gene distribution analysis and generation of TprA2/TprA gene tree

To identify genes that are highly enriched within the PMEN1 lineage relative to other pneumococcal lineages we clustered the coding sequences from 60 highly curated pneumococcal whole genome sequences (WGS), and selected clusters unique to the PMEN1 genomes. The 60 genomes are listed in S1 Table and marked as “To establish PMEN1 enrichment”, and the analysis has been previously described in detail [27]. Briefly, it involved CDS prediction by RAST [26], CDS clustering by utilizing tfasty36 (FASTA v.3.6 package) [70] and parsing the output to assemble genes that share at least 70% identity over 70% of their length into clusters of homologous sequences, and selecting clusters that are present in all PMEN1 genomes while absent in all other lineages.

To establish the gene presence/absence profiles within the 215 PMEN1 WGSs we performed an *in silico* PCR on the genomes previously published by Croucher and colleagues at the Sanger Center (listed in S1 Table [71]). In cases where the *in silico* analysis was inconclusive, we performed experimental PCR using forward and reverse primers to *tprA2*. To establish the gene presence/absence profiles within the 55 Streptococcal WGSs (S1 Table, strains labeled as “Distribution within Streptococcus sp.”), as displayed in Fig 1B, we employed the basic local alignment search tool (Blastn) using an e-value threshold of 1e-20 [72]. All of the *tprA2* CDSs displayed > = 95% similarity. The Lan locus is represented by three CDSs downstream of TprA2/PhrA2, and the Lan* locus is represented by seven CDSs downstream of TprA/PhrA; the genes with Lan and Lan* display exactly the same phylogenetic distribution in the 55 samples (i.e all present or all absent). In the vast majority of the genomes, the lantibiotic genes were neighboring the associated QS systems; the exceptions are genomes with contig breaks or low sequence coverage in these regions (these are noted in Fig 2).

The phylogenetic tree of *tprA2/tprA* was generated on the 48 sequences extracted in the analysis of the 55 streptococcal genomes. The nucleotide sequences were aligned using MAFFT (G-INS-i), and model selection was performed using MODELTEST. The phylogenetic tree was built with PhyML 3.0, model HKY+I(0.39) using maximum likelihood and 100 bootstrap replicates. Logos were generated from the C-terminal heptapeptides of (i) 6 PhrA2 sequences and (ii) 36 PhrA peptides using WebLogo [73] (Fig 9A and 9B).

## Supporting information

S1 Fig PCR performed on cDNA and genomic DNA to demonstrate transcriptional units. Lanes 1–4 are PCRs on cDNA template, lanes 5–8 on gDNA template. Primers used are as follows: lanes 1 and 5, *tprA2* fwd and *phrA2* rev; lanes 2 and 6, *lanA* fwd and *lanT* rev; lanes 3 and 7, *phrA2* fwd and *ABCATpase* rev; lanes 4 and 8 *gapdh*. Colored arrow heads in the genomic locus schematic indicate the primer binding sites corresponding to the bands on the gel (marked with the equivalent color). Prior to cDNA synthesis, all RNA samples were DNase-treated and subjected to a PCR check using primers for *gapdh* gene to ensure total elimination of DNA. Only when no amplification was observed in the gapdh check PCR was the cDNA synthesized.
(TIF)

S2 Fig PhrA2 modulates the expression levels of the TprA2 regulon. qRT-PCR measurements in gene expression of QS-lcp genes in WT strain PN4595-T23. Data was normalized to 16S rRNA expression. Y-axis displays fold change in gene expression, upon exposure to supernatant from Δ*tprA2* cultures or synthetic PhrA2, relative to media-only control. Error bars represent standard deviations for biological replicates (n = 3). Mid-log WT cells where split into three groups, and were submitted to treatment with media alone, cell-free supernatant from Δ*tprA2* cultures or, as a positive control, PhrA2 C-terminal heptapeptide (VDLGLAD). On the left, dark bars represent the fold change between addition of cell-free supernatant from Δ*tprA2* cultures relative to addition of media only. On the right side, striped bars represent the fold change between addition of PhrA2 C-terminal heptapeptide (VDLGLAD) relative to addition of media only. Both the culture supernatant and the PhrA2 heptapeptide lead to upregulation of *phrA2* and *lcpA*. * Statistically significant difference in gene expression (*P*-value<0.05).
(TIF)

S3 Fig *In vitro* condition where expression of PMEN1-*phrA2* and PMEN1-*phrA* is higher than that of D39-*phrA*. qRT-PCR measurement of cultures of PMEN1 and D39 grown independently in rich media (Columbia broth) to mid-log phase (n = 2). Statistical tests for gene expression: ‘**’*P*-value = 0.006 and ‘&’ *P*-value = 0.057.
(TIF)

S1 Table *S*. *pneumoniae* strains utilized for pangenome analysis. Bold: PMEN1 strains.
(PDF)

S2 Table Genes in this study. (PDF)

S3 Table *In vivo* phenotype of PN4595-T23 WT and isogenic mutants in a chinchilla model of pneumococcal disease. (PDF)

S4 Table PCR primers and nanoString probes used in the study. (PDF)

S5 Table List of genes with at least 2 fold difference in expression levels between wild-type PN4595-T23 and the isogenic Δ*tprA2* strain. (PDF)

## References

[1] O’Brien KL, Wolfson LJ, Watt JP, Henkle E, Deloria-Knoll M, McCall N, et al Burden of disease caused by Streptococcus pneumoniae in children younger than 5 years: global estimates. The Lancet. 2009;374: 893–902. 10.1016/S0140-6736(09)61204-6 19748398

[2] De Lencastre H, Tomasz A. From ecological reservoir to disease: the nasopharynx, day-care centres and drug-resistant clones of Streptococcus pneumoniae. J Antimicrob Chemother. 2002;50 Suppl S2: 75–81. 12556436 10.1093/jac/dkf511

[3] Kang L-H, Liu M-J, Xu W-C, Cui J-J, Zhang X-M, Wu K-F, et al Molecular epidemiology of pneumococcal isolates from children in China. Saudi Med J. 2016;37: 403–413. 10.15537/smj.2016.4.14507 27052283 PMC4852018

[4] Kadioglu A, Weiser JN, Paton JC, Andrew PW. The role of Streptococcus pneumoniae virulence factors in host respiratory colonization and disease. Nat Rev Microbiol. 2008;6: 288–301. 10.1038/nrmicro1871 18340341

[5] Branger S, Casalta JP, Habib G, Collard F, Raoult D. Streptococcus pneumoniae Endocarditis: Persistence of DNA on Heart Valve Material 7 Years after Infectious Episode. J Clin Microbiol. 2003;41: 4435–4437. 10.1128/JCM.41.9.4435-4437.2003 12958286 PMC193805

[6] Brown AO, Mann B, Gao G, Hankins JS, Humann J, Giardina J, et al Streptococcus pneumoniae Translocates into the Myocardium and Forms Unique Microlesions That Disrupt Cardiac Function. PLOS Pathog. 2014;10: e1004383 10.1371/journal.ppat.1004383 25232870 PMC4169480

[7] Forbes ML, Horsey E, Hiller NL, Buchinsky FJ, Hayes JD, Compliment JM, et al Strain-Specific Virulence Phenotypes of Streptococcus pneumoniae Assessed Using the Chinchilla laniger Model of Otitis Media. Ahmed N, editor. PLoS ONE. 2008;3: e1969 10.1371/journal.pone.0001969 18398481 PMC2279396

[8] Donati C, Hiller NL, Tettelin H, Muzzi A, Croucher NJ, Angiuoli SV, et al Structure and dynamics of the pan-genome of Streptococcus pneumoniae and closely related species. Genome Biol. 2010;11: R107 10.1186/gb-2010-11-10-r107 21034474 PMC3218663

[9] Hiller NL, Janto B, Hogg JS, Boissy R, Yu S, Powell E, et al Comparative genomic analyses of seventeen Streptococcus pneumoniae strains: insights into the pneumococcal supragenome. J Bacteriol. 2007;189: 8186–8195. 10.1128/JB.00690-07 17675389 PMC2168654

[10] McGee L, McDougal L, Zhou J, Spratt BG, Tenover FC, George R, et al Nomenclature of major antimicrobial-resistant clones of Streptococcus pneumoniae defined by the pneumococcal molecular epidemiology network. J Clin Microbiol. 2001;39: 2565–2571. 10.1128/JCM.39.7.2565-2571.2001 11427569 PMC88185

[11] Pletz MWR, McGee L, Jorgensen J, Beall B, Facklam RR, Whitney CG, et al Levofloxacin-resistant invasive Streptococcus pneumoniae in the United States: evidence for clonal spread and the impact of conjugate pneumococcal vaccine. Antimicrob Agents Chemother. 2004;48: 3491–3497. 10.1128/AAC.48.9.3491-3497.2004 15328116 PMC514755

[12] Reinert RR, Ringelstein A, van der Linden M, Cil MY, Al-Lahham A, Schmitz F-J. Molecular epidemiology of macrolide-resistant Streptococcus pneumoniae isolates in Europe. J Clin Microbiol. 2005;43: 1294–1300. 10.1128/JCM.43.3.1294-1300.2005 15750098 PMC1081259

[13] Croucher NJ, Harris SR, Fraser C, Quail MA, Burton J, van der Linden M, et al Rapid pneumococcal evolution in response to clinical interventions. Science. 2011;331: 430–434. 10.1126/science.1198545 21273480 PMC3648787

[14] Wyres KL, Lambertsen LM, Croucher NJ, McGee L, von Gottberg A, Liñares J, et al The multidrug-resistant PMEN1 pneumococcus is a paradigm for genetic success. Genome Biol. 2012;13: R103 10.1186/gb-2012-13-11-r103 23158461 PMC3580495

[15] Chancey ST, Agrawal S, Schroeder MR, Farley MM, Tettelin H, Stephens DS. Composite mobile genetic elements disseminating macrolide resistance in Streptococcus pneumoniae. Antimicrob Resist Chemother. 2015;6: 26. 10.3389/fmicb.2015.00026 PMC4321634 25709602

[16] Croucher NJ, Walker D, Romero P, Lennard N, Paterson GK, Bason NC, et al Role of conjugative elements in the evolution of the multidrug-resistant pandemic clone Streptococcus pneumoniaeSpain23F ST81. J Bacteriol. 2009;191: 1480–1489. 10.1128/JB.01343-08 19114491 PMC2648205

[17] Galperin MY, Nikolskaya AN, Koonin EV. Novel domains of the prokaryotic two-component signal transduction systems. FEMS Microbiol Lett. 2001;203: 11–21. 11557134 10.1111/j.1574-6968.2001.tb10814.x

[18] Rocha-Estrada J, Aceves-Diez AE, Guarneros G, de la Torre M. The RNPP family of quorum-sensing proteins in Gram-positive bacteria. Appl Microbiol Biotechnol. 2010;87: 913–923. 10.1007/s00253-010-2651-y 20502894

[19] Agaisse H, Gominet M, Økstad OA, Kolstø A-B, Lereclus D. PlcR is a pleiotropic regulator of extracellular virulence factor gene expression in Bacillus thuringiensis. Mol Microbiol. 1999;32: 1043–1053. 10361306 10.1046/j.1365-2958.1999.01419.x

[20] Gohar M, Faegri K, Perchat S, Ravnum S, Økstad OA, Gominet M, et al The PlcR Virulence Regulon of Bacillus cereus. PLoS ONE. 2008;3: e2793 10.1371/journal.pone.0002793 18665214 PMC2464732

[21] Riedel CU, Monk IR, Casey PG, Waidmann MS, Gahan CGM, Hill C. AgrD-dependent quorum sensing affects biofilm formation, invasion, virulence and global gene expression profiles in Listeria monocytogenes. Mol Microbiol. 2009;71: 1177–1189. 10.1111/j.1365-2958.2008.06589.x 19154329

[22] Lee MS, Morrison DA. Identification of a New Regulator in Streptococcus pneumoniae Linking Quorum Sensing to Competence for Genetic Transformation. J Bacteriol. 1999;181: 5004–5016. 10438773 10.1128/jb.181.16.5004-5016.1999 PMC93990

[23] Dawid S, Roche AM, Weiser JN. The blp Bacteriocins of Streptococcus pneumoniae Mediate Intraspecies Competition both In Vitro and In Vivo. Infect Immun. 2007;75: 443–451. 10.1128/IAI.01775-05 17074857 PMC1828380

[24] Hoover SE, Perez AJ, Tsui H-CT, Sinha D, Smiley DL, DiMarchi RD, et al A new quorum-sensing system (TprA/PhrA) for Streptococcus pneumoniae D39 that regulates a lantibiotic biosynthesis gene cluster. Mol Microbiol. 2015; 10.1111/mmi.13029 PMC4676566 25869931

[25] Paixão L, Oliveira J, Veríssimo A, Vinga S, Lourenço EC, Ventura MR, et al Host glycan sugar-specific pathways in Streptococcus pneumonia: galactose as a key sugar in colonisation and infection. PloS One. 2015;10: e0121042 10.1371/journal.pone.0121042 25826206 PMC4380338

[26] Overbeek R, Olson R, Pusch GD, Olsen GJ, Davis JJ, Disz T, et al The SEED and the Rapid Annotation of microbial genomes using Subsystems Technology (RAST). Nucleic Acids Res. 2014;42: D206–D214. 10.1093/nar/gkt1226 24293654 PMC3965101

[27] Eutsey RA, Powell E, Dordel J, Salter SJ, Clark TA, Korlach J, et al Genetic Stabilization of the Drug-Resistant PMEN1 Pneumococcus Lineage by Its Distinctive DpnIII Restriction-Modification System. mBio. 2015;6: e00173–15. 10.1128/mBio.00173-15 26081630 PMC4471560

[28] Li B, Yu JPJ, Brunzelle JS, Moll GN, van der Donk WA, Nair SK. Structure and mechanism of the lantibiotic cyclase involved in nisin biosynthesis. Science. 2006;311: 1464–1467. 10.1126/science.1121422 16527981

[29] Zhang Q, Yu Y, Vélasquez JE, van der Donk WA. Evolution of lanthipeptide synthetases. Proc Natl Acad Sci. 2012;109: 18361–18366. 10.1073/pnas.1210393109 23071302 PMC3494888

[30] Marchler-Bauer A, Lu S, Anderson JB, Chitsaz F, Derbyshire MK, DeWeese-Scott C, et al CDD: a Conserved Domain Database for the functional annotation of proteins. Nucleic Acids Res. 2011;39: D225–229. 10.1093/nar/gkq1189 21109532 PMC3013737

[31] Hiller NL, Eutsey RA, Powell E, Earl JP, Janto B, Martin DP, et al Differences in Genotype and Virulence among Four Multidrug-Resistant Streptococcus pneumoniae Isolates Belonging to the PMEN1 Clone. PLoS ONE. 2011;6: e28850 10.1371/journal.pone.0028850 22205975 PMC3242761

[32] LeMessurier KS, Ogunniyi AD, Paton JC. Differential expression of key pneumococcal virulence genes in vivo. Microbiology. 2006;152: 305–311. 10.1099/mic.0.28438-0 16436418

[33] Rutherford ST, Bassler BL. Bacterial Quorum Sensing: Its Role in Virulence and Possibilities for Its Control. Cold Spring Harb Perspect Med. 2012;2: a012427 10.1101/cshperspect.a012427 23125205 PMC3543102

[34] Kadam A, Janto B, Eutsey R, Earl JP, Powell E, Dahlgren ME, et al Streptococcus pneumoniae Supragenome Hybridization Arrays for Profiling of Genetic Content and Gene Expression. Curr Protoc Microbiol. 2015;36: 9D.4.1–9D.4.20. 25641101 10.1002/9780471729259.mc09d04s36 PMC4388488

[35] Bouillaut L, Perchat S, Arold S, Zorrilla S, Slamti L, Henry C, et al Molecular basis for group-specific activation of the virulence regulator PlcR by PapR heptapeptides. Nucleic Acids Res. 2008;36: 3791–3801. 10.1093/nar/gkn149 18492723 PMC2441798

[36] Rosch JW, Mann B, Thornton J, Sublett J, Tuomanen E. Convergence of Regulatory Networks on the Pilus Locus of Streptococcus pneumoniae. Infect Immun. 2008;76: 3187–3196. 10.1128/IAI.00054-08 18443093 PMC2446684

[37] Mann B, Opijnen, Wang J, Obert C, Wang Y-D, Carter R, et al Control of Virulence by Small RNAs in Streptococcus pneumoniae. PLOS Pathog. 2012;8: e1002788 10.1371/journal.ppat.1002788 22807675 PMC3395615

[38] Croucher NJ, Coupland PG, Stevenson AE, Callendrello A, Bentley SD, Hanage WP. Diversification of bacterial genome content through distinct mechanisms over different timescales. Nat Commun. 2014;5. 10.1038/ncomms6471 PMC4263131 25407023

[39] Valentino MD, McGuire AM, Rosch JW, Bispo PJM, Burnham C, Sanfilippo CM, et al Unencapsulated Streptococcus pneumoniae from conjunctivitis encode variant traits and belong to a distinct phylogenetic cluster. Nat Commun. 2014;5: 5411 10.1038/ncomms6411 25388376 PMC4231546

[40] Carvalho SM, Kloosterman TG, Kuipers OP, Neves AR. CcpA Ensures Optimal Metabolic Fitness of Streptococcus pneumoniae. PLoS ONE. 2011;6: e26707 10.1371/journal.pone.0026707 22039538 PMC3198803

[41] Corso A, Severina EP, Petruk VF, Mauriz YR, Tomasz A. Molecular characterization of penicillin-resistant Streptococcus pneumoniae isolates causing respiratory disease in the United States. Microb Drug Resist Larchmt N. 1998;4: 325–337. 10.1089/mdr.1998.4.325 9988052

[42] Muñoz R, Coffey TJ, Daniels M, Dowson CG, Laible G, Casal J, et al Intercontinental spread of a multiresistant clone of serotype 23F Streptococcus pneumoniae. J Infect Dis. 1991;164: 302–306. 1856478 10.1093/infdis/164.2.302

[43] Roberts RB, Tomasz A, Corso A, Hargrave J, Severina E, PRP Collaborative Study Group. Penicillin-resistant Streptococcus pneumoniae in metropolitan New York hospitals: case control study and molecular typing of resistant isolates. Microb Drug Resist Larchmt N. 2001;7: 137–152. 10.1089/10766290152045011 11442340

[44] Brueggemann AB, Griffiths DT, Meats E, Peto T, Crook DW, Spratt BG. Clonal relationships between invasive and carriage Streptococcus pneumoniae and serotype- and clone-specific differences in invasive disease potential. J Infect Dis. 2003;187: 1424–1432. 10.1086/374624 12717624

[45] Hanage WP, Kaijalainen TH, Syrjänen RK, Auranen K, Leinonen M, Mäkelä PH, et al Invasiveness of Serotypes and Clones of Streptococcus pneumoniae among Children in Finland. Infect Immun. 2005;73: 431–435. 10.1128/IAI.73.1.431-435.2005 15618181 PMC538975

[46] Zemlickova H, Jakubu V, Urbaskova P, Motlova J, Musilek M, Adamkova V. Serotype-specific invasive disease potential of Streptococcus pneumoniae in Czech children. J Med Microbiol. 2010;59: 1079–1083. 10.1099/jmm.0.018390-0 20508002

[47] Sjöström K, Spindler C, Ortqvist A, Kalin M, Sandgren A, Kühlmann-Berenzon S, et al Clonal and capsular types decide whether pneumococci will act as a primary or opportunistic pathogen. Clin Infect Dis Off Publ Infect Dis Soc Am. 2006;42: 451–459. 10.1086/499242 16421787

[48] Parashar V, Aggarwal C, Federle MJ, Neiditch MB. Rgg protein structure-function and inhibition by cyclic peptide compounds. Proc Natl Acad Sci U S A. 2015;112: 5177–5182. 10.1073/pnas.1500357112 25847993 PMC4413276

[49] Aggarwal C, Jimenez JC, Nanavati D, Federle MJ. Multiple length peptide-pheromone variants produced by Streptococcus pyogenes directly bind Rgg proteins to confer transcriptional regulation. J Biol Chem. 2014;289: 22427–22436. 10.1074/jbc.M114.583989 24958729 PMC4139249

[50] Chang JC, LaSarre B, Jimenez JC, Aggarwal C, Federle MJ. Two Group A Streptococcal Peptide Pheromones Act through Opposing Rgg Regulators to Control Biofilm Development. PLOS Pathog. 2011;7: e1002190 10.1371/journal.ppat.1002190 21829369 PMC3150281

[51] Frazão N, Hiller NL, Powell E, Earl J, Ahmed A, Sá-Leão R, et al Virulence potential and genome-wide characterization of drug resistant Streptococcus pneumoniae clones selected in vivo by the 7-valent pneumococcal conjugate vaccine. PloS One. 2013;8: e74867 10.1371/journal.pone.0074867 24069360 PMC3777985

[52] Keller LE, Thomas JC, Luo X, Nahm MH, McDaniel LS, Robinson DA. Draft Genome Sequences of Five Multilocus Sequence Types of Nonencapsulated Streptococcus pneumoniae. Genome Announc. 2013;1. 10.1128/genomeA.00520-13 PMC3735068 23887920

[53] Zomer AL, Buist G, Larsen R, Kok J, Kuipers OP. Time-resolved determination of the CcpA regulon of Lactococcus lactis subsp. cremoris MG1363. J Bacteriol. 2007;189: 1366–1381. 10.1128/JB.01013-06 17028270 PMC1797362

[54] Al-Bayati FAY, Kahya HFH, Damianou A, Shafeeq S, Kuipers OP, Andrew PW, et al Pneumococcal galactose catabolism is controlled by multiple regulators acting on pyruvate formate lyase. Sci Rep. 2017;7: 43587 10.1038/srep43587 28240278 PMC5327383

[55] Zähner D, Hakenbeck R. The Streptococcus pneumoniae Beta-Galactosidase Is a Surface Protein. J Bacteriol. 2000;182: 5919–5921. 11004197 10.1128/jb.182.20.5919-5921.2000 PMC94720

[56] Baldi P, Long AD. A Bayesian framework for the analysis of microarray expression data: regularized t -test and statistical inferences of gene changes. Bioinforma Oxf Engl. 2001;17: 509–519. 10.1093/bioinformatics/17.6.509 11395427

[57] Kayala MA, Baldi P. Cyber-T web server: differential analysis of high-throughput data. Nucleic Acids Res. 2012;40: W553–559. 10.1093/nar/gks420 22600740 PMC3394347

[58] Ruijter JM, Lorenz P, Tuomi JM, Hecker M, van den Hoff MJB. Fluorescent-increase kinetics of different fluorescent reporters used for qPCR depend on monitoring chemistry, targeted sequence, type of DNA input and PCR efficiency. Mikrochim Acta. 2014;181: 1689–1696. 10.1007/s00604-013-1155-8 25253910 PMC4167442

[59] Ramakers C, Ruijter JM, Deprez RHL, Moorman AFM. Assumption-free analysis of quantitative real-time polymerase chain reaction (PCR) data. Neurosci Lett. 2003;339: 62–66. 12618301 10.1016/s0304-3940(02)01423-4

[60] Xu W, Solis NV, Filler SG, Mitchell AP. Pathogen Gene Expression Profiling During Infection Using a Nanostring nCounter Platform. Methods Mol Biol Clifton NJ. 2016;1361: 57–65. 10.1007/978-1-4939-3079-1_3 PMC5317040 26483015

[61] Kim W, Park HK, Hwang W-J, Shin H-S. Simultaneous Detection of Streptococcus pneumoniae, S. mitis, and S. oralis by a Novel Multiplex PCR Assay Targeting the gyrB Gene. J Clin Microbiol. 2013;51: 835–840. 10.1128/JCM.02920-12 23269740 PMC3592088

[62] Terrasse R, Tacnet-Delorme P, Moriscot C, Pérard J, Schoehn G, Vernet T, et al Human and Pneumococcal Cell Surface Glyceraldehyde-3-phosphate Dehydrogenase (GAPDH) Proteins Are Both Ligands of Human C1q Protein. J Biol Chem. 2012;287: 42620–42633. 10.1074/jbc.M112.423731 23086952 PMC3522263

[63] Buchinsky FJ, Forbes ML, Hayes JD, Shen K, Ezzo S, Compliment J, et al Virulence phenotypes of low-passage clinical isolates of nontypeable Haemophilus influenzae assessed using the chinchilla laniger model of otitis media. BMC Microbiol. 2007;7: 56 10.1186/1471-2180-7-56 17570853 PMC1914350

[64] Zbinden A, Mueller NJ, Tarr PE, Eich G, Schulthess B, Bahlmann AS, et al Streptococcus tigurinus, a novel member of the Streptococcus mitis group, causes invasive infections. J Clin Microbiol. 2012;50: 2969–2973. 10.1128/JCM.00849-12 22760039 PMC3421813

[65] Darling ACE, Mau B, Blattner FR, Perna NT. Mauve: Multiple Alignment of Conserved Genomic Sequence With Rearrangements. Genome Res. 2004;14: 1394–1403. 10.1101/gr.2289704 15231754 PMC442156

[66] Darling AE, Mau B, Perna NT. progressiveMauve: Multiple Genome Alignment with Gene Gain, Loss and Rearrangement. PLoS ONE. 2010;5: e11147 10.1371/journal.pone.0011147 20593022 PMC2892488

[67] Katoh K, Misawa K, Kuma K, Miyata T. MAFFT: a novel method for rapid multiple sequence alignment based on fast Fourier transform. Nucleic Acids Res. 2002;30: 3059–3066. 12136088 10.1093/nar/gkf436 PMC135756

[68] Posada D, Crandall KA. MODELTEST: testing the model of DNA substitution. Bioinformatics. 1998;14: 817–818. 9918953 10.1093/bioinformatics/14.9.817

[69] Guindon S, Dufayard J-F, Lefort V, Anisimova M, Hordijk W, Gascuel O. New algorithms and methods to estimate maximum-likelihood phylogenies: assessing the performance of PhyML 3.0. Syst Biol. 2010;59: 307–321. 10.1093/sysbio/syq010 20525638

[70] Pearson WR, Lipman DJ. Improved tools for biological sequence comparison. Proc Natl Acad Sci U S A. 1988;85: 2444–2448. 3162770 10.1073/pnas.85.8.2444 PMC280013

[71] Croucher NJ, Harris SR, Fraser C, Quail MA, Burton J, van der Linden M, et al Rapid pneumococcal evolution in response to clinical interventions. Science. 2011;331: 430–434. 10.1126/science.1198545 21273480 PMC3648787

[72] Altschul SF, Gish W, Miller W, Myers EW, Lipman DJ. Basic local alignment search tool. J Mol Biol. 1990;215: 403–410. 10.1016/S0022-2836(05)80360-2 2231712

[73] Crooks GE, Hon G, Chandonia J-M, Brenner SE. WebLogo: a sequence logo generator. Genome Res. 2004;14: 1188–1190. 10.1101/gr.849004 15173120 PMC419797

