# Supplementary figures and images for: Promiscuous signaling by a regulatory system unique to the pandemic PMEN1 pneumococcal lineage
Source: PLoS Pathog. 2017 May 18;13(5):e1006339. doi: 10.1371/journal.ppat.1006339 (PMC5436883; doi:10.1371/journal.ppat.1006339)

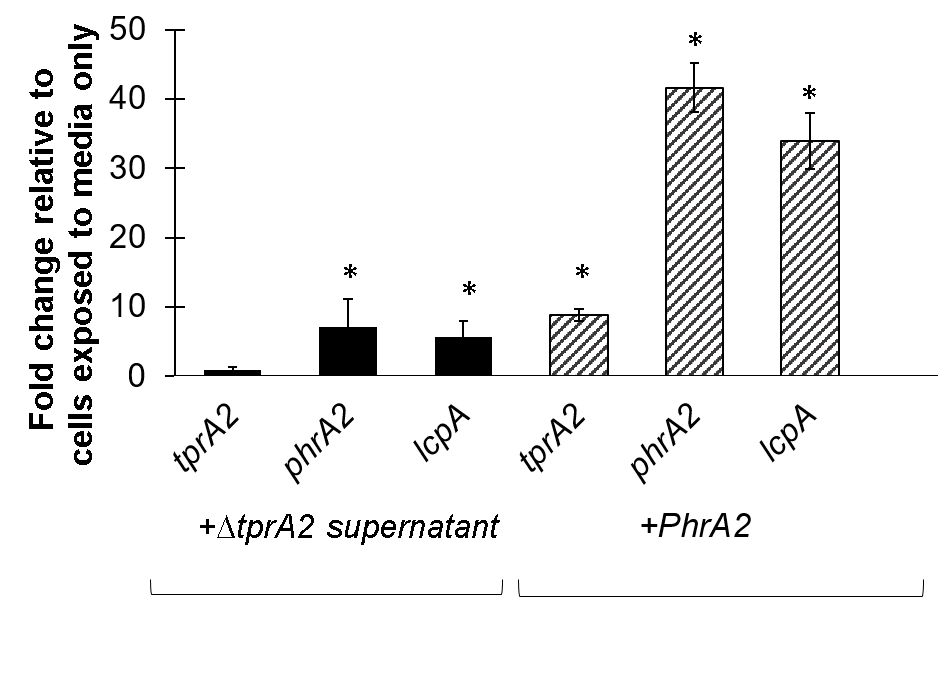

Supplement: S2 Fig — qRT-PCR measurements in gene expression of QS-lcp genes in WT strain PN4595-T23. Data was normalized to 16S rRNA expression. Y-axis displays fold change in gene expression, upon exposure to supernatant from ΔtprA2 cultures or synthetic PhrA2, relative to media-only control. Error bars represent standard deviations for biological replicates (n = 3). Mid-log WT cells where split into three groups, and were submitted to treatment with media alone, cell-free supernatant from ΔtprA2 cultures or, as a positive control, PhrA2 C-terminal heptapeptide (VDLGLAD). On the left, dark bars represent the fold change between addition of cell-free supernatant from ΔtprA2 cultures relative to addition of media only. On the right side, striped bars represent the fold change between addition of PhrA2 C-terminal heptapeptide (VDLGLAD) relative to addition of media only. Both the culture supernatant and the PhrA2 heptapeptide lead to upregulation of phrA2 and lcpA. * Statistically significant difference in gene expression (P-value<0.05). (TIF) [file ppat.1006339.s002.tif]

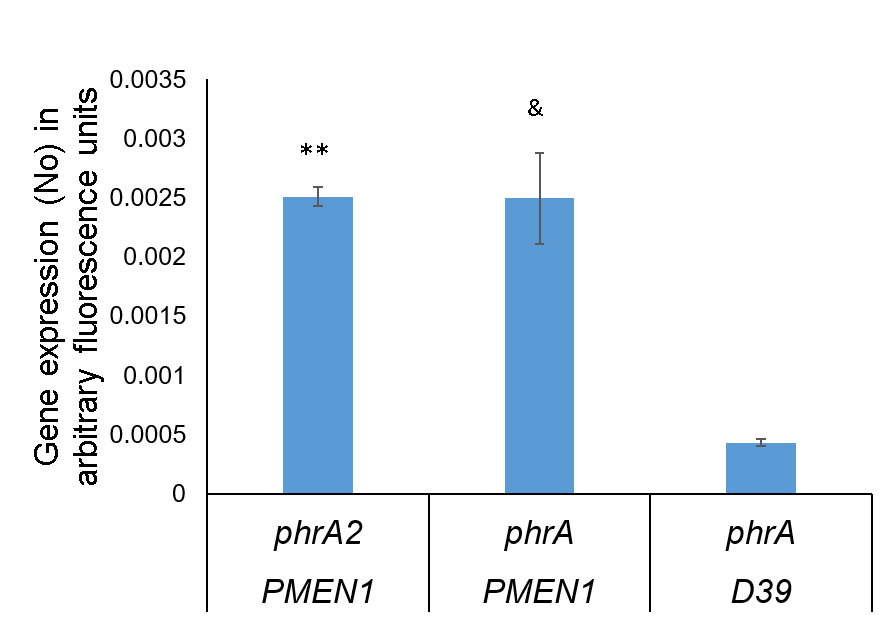

Supplement: S3 Fig — qRT-PCR measurement of cultures of PMEN1 and D39 grown independently in rich media (Columbia broth) to mid-log phase (n = 2). Statistical tests for gene expression: ‘**’P-value = 0.006 and ‘&’ P-value = 0.057. (TIF) [file ppat.1006339.s003.tif]
