# Supplementary material for: Promiscuous signaling by a regulatory system unique to the pandemic PMEN1 pneumococcal lineage
Source: PLoS Pathog. 2017 May 18;13(5):e1006339. doi: 10.1371/journal.ppat.1006339 (PMC5436883; doi:10.1371/journal.ppat.1006339)
Supplement: S1 Table — Bold: PMEN1 strains. (PDF) [file ppat.1006339.s004.pdf]

S1 Table. *S. pneumoniae* strains utilized for pangenome analysis.Bold: PMEN1 strains.

Strain ID	Species	GenBank Accession No.	Analyses performed	
SK1076	*Streptococcus infantis*	AFNN00000000	Distribution within Streptococcus sp.	
SK970	*Streptococcus infantis*	AFUT00000000	Distribution within Streptococcus sp.	
ATCC 6249	*Streptococcus mitis*	AEEN00000000	Distribution within Streptococcus sp.	
B6	*Streptococcus mitis*	FN568063	Distribution within Streptococcus sp.	
bv. 2 str. F0392	*Streptococcus mitis*	AFUO00000000	Distribution within Streptococcus sp.	
bv. 2 str. SK95	*Streptococcus mitis*	AFUB00000000	Distribution within Streptococcus sp.	
NCTC 12261	*Streptococcus mitis*	AEDX00000000	Distribution within Streptococcus sp.	
SK1073	*Streptococcus mitis*	AFQT00000000	Distribution within Streptococcus sp.	
SK1080	*Streptococcus mitis*	AFQV00000000	Distribution within Streptococcus sp.	
SK321	*Streptococcus mitis*	AEDT00000000	Distribution within Streptococcus sp.	
SK564	*Streptococcus mitis*	AEDU00000000	Distribution within Streptococcus sp.	
SK569	*Streptococcus mitis*	AFUF00000000	Distribution within Streptococcus sp.	
SK575	*Streptococcus mitis*	AICU01000001	Distribution within Streptococcus sp.	
SK597	*Streptococcus mitis*	AEDV00000000	Distribution within Streptococcus sp.	
Uo5	*Streptococcus oralis*	FR720602	Distribution within Streptococcus sp.	
**PN4595-T23**	*Streptococcus pneumoniae*	ABXO00000000	Distribution within Streptococcus sp.	To establish PMEN1 enrichment
70585	*Streptococcus pneumoniae*	CP000918	Distribution within Streptococcus sp.	To establish PMEN1 enrichment
**ATCC 700669**	*Streptococcus pneumoniae*	FM211187	Distribution within Streptococcus sp.	To establish PMEN1 enrichment
CDC0288_04	*Streptococcus pneumoniae*	ABGF00000000	Distribution within Streptococcus sp.	To establish PMEN1 enrichment
CDC1087-00	*Streptococcus pneumoniae*	ABFT00000000	Distribution within Streptococcus sp.	To establish PMEN1 enrichment
CDC1873-00	*Streptococcus pneumoniae*	ABFS00000000	Distribution within Streptococcus sp.	To establish PMEN1 enrichment
CDC3059-06	*Streptococcus pneumoniae*	ABGG00000000	Distribution within Streptococcus sp.	To establish PMEN1 enrichment
CGSP14	*Streptococcus pneumoniae*	CP001033	Distribution within Streptococcus sp.	To establish PMEN1 enrichment
D39	*Streptococcus pneumoniae*	CP000410	Distribution within Streptococcus sp.	To establish PMEN1 enrichment
G54	*Streptococcus pneumoniae*	CP001015	Distribution within Streptococcus sp.	To establish PMEN1 enrichment
GA13494	*Streptococcus pneumoniae*	AGOZ01000001	Distribution within Streptococcus sp.	To establish PMEN1 enrichment
Hungary19A-6	*Streptococcus pneumoniae*	CP000936	Distribution within Streptococcus sp.	To establish PMEN1 enrichment
ICE44	*Streptococcus pneumoniae*	AUYF00000000	Distribution within Streptococcus sp.	To establish PMEN1 enrichment
INV104	*Streptococcus pneumoniae*	FQ312030	Distribution within Streptococcus sp.	To establish PMEN1 enrichment
JJA	*Streptococcus pneumoniae*	CP000919	Distribution within Streptococcus sp.	To establish PMEN1 enrichment
MNZ14	*Streptococcus pneumoniae*	ASJO00000000	Distribution within Streptococcus sp.	
MNZ85	*Streptococcus pneumoniae*	ASJF00000000	Distribution within Streptococcus sp.	
SP11-BS70	*Streptococcus pneumoniae*	ABAC00000000	Distribution within Streptococcus sp.	To establish PMEN1 enrichment
SP14-BS69	*Streptococcus pneumoniae*	ABAD00000000	Distribution within Streptococcus sp.	To establish PMEN1 enrichment
SP18-BS74	*Streptococcus pneumoniae*	ABAE00000000	Distribution within Streptococcus sp.	To establish PMEN1 enrichment
SP19-BS75	*Streptococcus pneumoniae*	ABAF00000000	Distribution within Streptococcus sp.	To establish PMEN1 enrichment

SP23-BS72	*Streptococcus pneumoniae*	ABAG00000000	Distribution within Streptococcus sp.	To establish PMEN1 enrichment
SP3-BS71	*Streptococcus pneumoniae*	AAZZ00000000	Distribution within Streptococcus sp.	To establish PMEN1 enrichment
SP6-BS73	*Streptococcus pneumoniae*	ABAA00000000	Distribution within Streptococcus sp.	To establish PMEN1 enrichment
SP9-BS68	*Streptococcus pneumoniae*	ABAB00000000	Distribution within Streptococcus sp.	To establish PMEN1 enrichment
SPN1041	*Streptococcus pneumoniae*	CACE00000000	Distribution within Streptococcus sp.	
SPNA45	*Streptococcus pneumoniae*	CACG00000000	Distribution within Streptococcus sp.	To establish PMEN1 enrichment
**SV35-T23**	*Streptococcus pneumoniae*	ADNN00000000	Distribution within Streptococcus sp.	To establish PMEN1 enrichment
**SV36-T3**	*Streptococcus pneumoniae*	ADNO00000000	Distribution within Streptococcus sp.	To establish PMEN1 enrichment
Taiwan19F-14	*Streptococcus pneumoniae*	CP000921	Distribution within Streptococcus sp.	To establish PMEN1 enrichment
TIGR4	*Streptococcus pneumoniae*	AE005672	Distribution within Streptococcus sp.	To establish PMEN1 enrichment
WL400	*Streptococcus pneumoniae*	AVFA00000000	Distribution within Streptococcus sp.	
WL677	*Streptococcus pneumoniae*	AUWZ00000000	Distribution within Streptococcus sp.	
ATCC BAA-960	*Streptococcus pseudopneumoniae*	AICS00000000	Distribution within Streptococcus sp.	
IS7493	*Streptococcus pseudopneumoniae*	CP002925	Distribution within Streptococcus sp.	
SK674	*Streptococcus pseudopneumoniae*	AJKE00000000	Distribution within Streptococcus sp.	
2426	*Streptococcus tigurinus*	ASXA00000000	Distribution within Streptococcus sp.	
AZ_3a	*Streptococcus tigurinus*	AORU00000000	Distribution within Streptococcus sp.	
INV200	*Streptococcus pneumoniae*	FQ312029	Distribution within pneumococcus	To establish PMEN1 enrichment
ST13v1	*Streptococcus pneumoniae*	NZ_ABWQ00000000	Distribution within pneumococcus	To establish PMEN1 enrichment
ST13v12	*Streptococcus pneumoniae*	NZ_ABWU00000000	Distribution within pneumococcus	To establish PMEN1 enrichment
ST13v6	*Streptococcus pneumoniae*	NZ_ABWB01000000	Distribution within pneumococcus	To establish PMEN1 enrichment
AP200	*Streptococcus pneumoniae*	CP002121	Distribution within pneumococcus	To establish PMEN1 enrichment
MLV016	*Streptococcus pneumoniae*	ABGH00000000	Distribution within pneumococcus	To establish PMEN1 enrichment
ICE59	*Streptococcus pneumoniae*	AUYE00000000	Distribution within pneumococcus	To establish PMEN1 enrichment
Sp647	*Streptococcus pneumoniae*	AUYL00000000	Distribution within pneumococcus	To establish PMEN1 enrichment
Sp6706B	*Streptococcus pneumoniae*	CP002176	Distribution within pneumococcus	To establish PMEN1 enrichment
SPAIN6B	*Streptococcus pneumoniae*	AUYK00000000	Distribution within pneumococcus	To establish PMEN1 enrichment
CCRI_1974M2	*Streptococcus pneumoniae*	ABZT00000000	Distribution within pneumococcus	To establish PMEN1 enrichment
CCRI_1974	*Streptococcus pneumoniae*	ABZC00000000	Distribution within pneumococcus	To establish PMEN1 enrichment
SP195	*Streptococcus pneumoniae*	ABGE00000000	Distribution within pneumococcus	To establish PMEN1 enrichment
Spain1417	*Streptococcus pneumoniae*	AUYH00000000	Distribution within pneumococcus	To establish PMEN1 enrichment
Sp88-1	*Streptococcus pneumoniae*	AUYI00000000	Distribution within pneumococcus	To establish PMEN1 enrichment
Sp439-1	*Streptococcus pneumoniae*	AUYJ00000000	Distribution within pneumococcus	To establish PMEN1 enrichment
SPAIN9V	*Streptococcus pneumoniae*	AUYG00000000	Distribution within pneumococcus	To establish PMEN1 enrichment
N034156	*Streptococcus pneumoniae*	FQ312045	Distribution within pneumococcus	To establish PMEN1 enrichment
N034183	*Streptococcus pneumoniae*	FQ312043	Distribution within pneumococcus	To establish PMEN1 enrichment
N994038	*Streptococcus pneumoniae*	FQ312041	Distribution within pneumococcus	To establish PMEN1 enrichment
N994039	*Streptococcus pneumoniae*	FQ312044	Distribution within pneumococcus	To establish PMEN1 enrichment

OXC141	*Streptococcus pneumoniae*	FQ312027	Distribution within pneumococcus	To establish PMEN1 enrichment
SPN021198	*Streptococcus pneumoniae*	CACH01000000	Distribution within pneumococcus	To establish PMEN1 enrichment
SPN072838	*Streptococcus pneumoniae*	CACI01000000	Distribution within pneumococcus	To establish PMEN1 enrichment
SPN1041	*Streptococcus pneumoniae*	CACE01000000	Distribution within pneumococcus	To establish PMEN1 enrichment
P1031	*Streptococcus pneumoniae*	CP000920	Distribution within pneumococcus	To establish PMEN1 enrichment
N032672	*Streptococcus pneumoniae*	FQ312039	Distribution within pneumococcus	To establish PMEN1 enrichment
N033038	*Streptococcus pneumoniae*	FQ312042	Distribution within pneumococcus	To establish PMEN1 enrichment
SPN061370	*Streptococcus pneumoniae*	CACJ01000000	Distribution within pneumococcus	To establish PMEN1 enrichment
R6	*Streptococcus pneumoniae*	AE007317	Distribution within pneumococcus	To establish PMEN1 enrichment
N7465	*Streptococcus pneumoniae*	CACF01000000	Distribution within pneumococcus	To establish PMEN1 enrichment
ST2011v4	*Streptococcus pneumoniae*	NZ_ADHN01000000	Distribution within pneumococcus	To establish PMEN1 enrichment
