# Supplementary material for: Promiscuous signaling by a regulatory system unique to the pandemic PMEN1 pneumococcal lineage
Source: PLoS Pathog. 2017 May 18;13(5):e1006339. doi: 10.1371/journal.ppat.1006339 (PMC5436883; doi:10.1371/journal.ppat.1006339)
Supplement: S2 Table — (PDF) [file ppat.1006339.s005.pdf]

S2 Table. Genes in this study

| Gene Name | Gene ID in PN4595-T23 | Gene ID in ATCC700669 | Annotation |
|---|---|---|---|
| *tprA2* | CGSSp4595_1262 | SPN23F_12750 | Transcriptional regulator from TprA2/PhrA2 system |
| *phrA2* | CGSSp4595_1261 | SPN23F_12740 | Signaling peptide from TprA2/PhrA2 system |
| ABC transporter ATPase | CGSSp4595_1260 | SPN23F_12730 | ABC transporter ATPase, same transcript as PhrA2 |
| ABC transporter permease | CGSSp4595_1259 | SPN23F_12720 | putative membrane protein |
| ABC transporter permease | CGSSp4595_1258 | SPN23F_12710 | putative ABC transporter, permease protein |
| *lcpA* | * | SPN23F_12701 | Lanthionine-containing peptide |
| *lcpM* | CGSSp4595_1257 | SPN23F_12700 | predicted LcpA modifying enzyme |
| *lcpT* | CGSSp4595_1256 | SPN23F_12690 | predicted LcpA transporter |
| *tprA* | CGSSp4595_1947 | SPN23F_19680 | helix-turn-helix family protein |
| *phrA* | CGSSp4595_1948 | SPN23F_19690 | Signaling peptide from TprA/PhrA system |
| LanA associated with TprA | CGSSp4595_1949 | SPN23F_19700 | conserved hypothetical protein |
| LanA associated with TprA | CGSSp4595_1950 | SPN23F_19710 | Type II Lantibiotic-like peptide |
| LanM associated with TprA | CGSSp4595_1951 | SPN23F_19720 | Lantibiotic synthetase |
| conserved hypothetical protein | CGSSp4595_1952 | SPN23F_19740 | conserved hypothetical protein |
| toxin secretion ABC transporter ATP-binding/permease protein | CGSSp4595_1953 | SPN23F_19750 | toxin secretion ABC transporter ATP-binding/permease protein |
| LanT associated with TprA | CGSSp4595_1954 | SPN23F_19750 | Lantibiotic transporter |

* not annotated in original genome submission
