# Supplementary material for: Promiscuous signaling by a regulatory system unique to the pandemic PMEN1 pneumococcal lineage
Source: PLoS Pathog. 2017 May 18;13(5):e1006339. doi: 10.1371/journal.ppat.1006339 (PMC5436883; doi:10.1371/journal.ppat.1006339)
Supplement: S3 Table — (PDF) [file ppat.1006339.s006.pdf]

Table S3. *In vivo* phenotype of PN4595-T23 WT and isogenic mutants in a chinchilla model of pneumococcal disease

	Strains & Phenotype			*P* -values		
Strain	PN4595-T23 (WT)	Δ*phrA2-ABC*	Δ*tprA2*	WT vs Δ*phrA2-ABC*	WT vs Δ*tprA2*	Δ*tprA2* vs Δ*phrA2-ABC*
Mortality	14/19 (74%)	7/10 (70%)	7/10 (70%)	NS	NS	NS
Bacteria in the Brain	18/19 (95%)	8/10 (80%)	8/10 (80%)	NS	NS	NS
Bacteria in the Lung	10/19 (52%)	2/10 (20%)	8/10 (80%)	0.048	NS	0.0041
