# Supplementary material for: Promiscuous signaling by a regulatory system unique to the pandemic PMEN1 pneumococcal lineage
Source: PLoS Pathog. 2017 May 18;13(5):e1006339. doi: 10.1371/journal.ppat.1006339 (PMC5436883; doi:10.1371/journal.ppat.1006339)
Supplement: S4 Table — (PDF) [file ppat.1006339.s007.pdf]

Table S4. PCR primers and nanoString probes used in the study.

**Pimer name**	**Primer Sequence**	**Application**
Sp23F_12750_Flank1F	ACCGACCAAATTCCCAACTATCTC	Deletion of *tprA2*
Sp23F_12750_Flank1R_XhoI	ATATATCTCGAGTGGTCATCTCTAACCCTCTTTAAA	Deletion of *tprA2*
Sp23F_12750_Flank2F_NheI	ATATATGCTAGCGATAGGTGACTTCGATTGTAATAAG	Deletion of *tprA2*
Sp23F_12750_Flank2R	CGGCGAAATATTTTAGTCGAACA	Deletion of *tprA2*
Sp23F_12750_F1SpecF2F	CCATGGGAATTAAGATACCACTACTCA	Deletion of *tprA2*
Sp23F_12750_F1SpecF2R	GAAATCCATCATGTGAGAAAACTAAAGG	Deletion of *tprA2*
F1_ermB_no_promo_fwd	GCGCTTCTATGATTTTCAG	Overexpression of *tprA2*
F1_ermB_no_promo_rev	aaccctctTTATTTCCTCCCGTTAAATAATAG	Overexpression of *tprA2*
plcR_no_promo_fwd	gaggaaataaAGAGGGTTAGAGATGACC	Overexpression of *tprA2*
plcR_no_promo_rev	taactttccTCACCTATCCTTATCTTTCAAAAAATG	Overexpression of *tprA2*
F2_no_promo_fwd	gataggtgaGGAAAGTTACACGTTACTAAAG	Overexpression of *tprA2*
F2_no_promo_rev	GCCCACGAGTAAAGAGAC	Overexpression of *tprA2*
F1_ermB_promo_fwd	CCCATTCATGCAGGAATTATG	Overexpression of *tprA2*
F1_ermB_promo_rev	tttctaatacTTATTTCCTCCCGTTAAATAATAG	Overexpression of *tprA2*
plcR_with_promo_fwd	gaggaaataaGTATTAGAAAGTTGAAAAATAGAGATTAAAAG	Overexpression of *tprA2*
plcR_with_promo_rev	taactttccTCACCTATCCTTATCTTTCAAAAAATG	Overexpression of *tprA2*
F2_promo_fwd	gataggtgaGGAAAGTTACACGTTACTAAAGG	Overexpression of *tprA2*
F2_promo_rev	GCTTGAGTTTGCCCACGA	Overexpression of *tprA2*
spec_fwd	aaatgagaaatccagGGATCCCCCGTTTGATTTTTAATG	For Δ*phrA2-ABC/OElanAMT*
spec_rev	aatgttcatcatgcgTGGATCCAATTTTTTTATAATTTTTTAATCTG	For Δ*phrA2-ABC/OElanAMT*
ABC_F2_fwd	aaaaaattggatccaCGCATGATGAACATTATTTTTG	For Δ*phrA2-ABC/OElanAMT*
ABC_F2_rev	CTCCAGTTTCACCTTTTAAC	For Δ*phrA2-ABC/OElanAMT*
Pep+ABC_F1_fwd	GCCATCATGAGCTTGTGTTC	For Δ*phrA2-ABC*
Pep+ABC_F1_rev	tcaaacgggggatccCTCATATTAACCACCTTTTTCTTATC	For Δ*phrA2-ABC/OElanAMT*
Spec_term_rev	agcggggtttttgcgTGGATCCAATTTTTTTATAATTTTTTAATCTG	For Δ*phrA2-ABC/OElanAMT::OEphrA2-ABC*
Pep+ABC_F1_fwd	GCCATCATGAGCTTGTGTTC	For Δ*phrA2-ABC/OElanAMT::OEphrA2-ABC*
Pep+ABC_F1_rev	tcaaacgggggatccCTCATATTAACCACCTTTTTCTTATC	For Δ*phrA2-ABC/OElanAMT::OEphrA2-ABC*
spec_fwd (+pep)	ggtggttaatatgagGGATCCCCCGTTTGATTTTTAATG	For Δ*phrA2-ABC/OElanAMT::OEphrA2-ABC*
Spec-F	ATCGATTTTCGTTCGTGAATACATGTTAT	For D39Δ*phrA2*
Spec-R	GTTATGCAAGGGTTTATTGTTTTCTA	For D39Δ*phrA2*
LF-SOE1745-F	GCGTCCAACGTGGCTCTGCACCA	For D39Δ*phrA2*
LF-SOE1745-R	TATTCACGAACGAAAATCGATTTTCTCTGCGAGTGTATTCATTA	For D39Δ*phrA2*
RF-SOE1745-F	AACAATAAACCCTTGCATAACAAGGAACTAGATACTGTTTAGTT	For D39Δ*phrA2*
RF-SOE1745-R	GCTAGGACACTATGGACTTCTTG	For D39Δ*phrA2*
**Nanostring Probe Name**		
tprA2	AGAAGCCGTCCAAGAAGCCTTAGAAACCATTGACTTTTGTAAACAAAAAGAGACCAGCTATCAGCTGGCTCCTCTTTTAACTATCGTTGCGAATGCCGGA	enumeration of *tprA2* mRNA transcripts in nCounter
ABC ATPase	GAGAAATCTATGGCTTACTAGGAAGAAATGGTACTGGAAAGACAACCTTTATCAAGGCTATTTTAGGCTTGACAGCTATGGATAGTGGTGAAGTGAATAT	enumeration of *phrA2* and *ABC ATPase* mRNA transcripts in nCounter
lcpA	AAAAAACCTATTTACGAATCACTAAGTACAGAAGGTAATCCAGCTGGGGATATCTTGCGTGAACTTAGCGATAGCGAGATGGATCAAGCTATGGCTGGCG	enumeration of *lcpA* mRNA transcripts in nCounter
lcpM	TTACCTAATATAGTTTGGGGTAAAGATGGGAACTCTGGGGTGGATATTAGCGGTCTAAGTTCAAGCGCAGGAGAGATGATTCCGATTGAACGTGCAAGTA	enumeration of *lcpM* mRNA transcripts in nCounter
lcpT	CAATTATCTGAGACCAGTTGTGGTTTAGCATGCATGGCAATGATTTTAGATTATTATGGGCATGAGGCAAACTTATATGAATTATGCTGTGATTTTGAGA	enumeration of *lcpT* mRNA transcripts in nCounter
gyrB	AAGGTTGGAAGCGAGATTAAAGAATATATCCAGCCGGGTGCAGATCAAGAAATCAAACTCCAAGAAGCTTTAGCCCGTTATAGTGAAGGTCGTACCAAAC	enumeration of gyrB mRNA transcripts in nCounter
metG	GGTCGCCTTAATGAAATGCTACGCAACTTCATCGAGCCAGGTTTGGAAGATTTGGCGGTATCTCGTACAACCTTTACATGGGGAGTGCCTGTCCCATCAA	enumeration of metG mRNA transcripts in nCounter
