# Supplementary material for: Promiscuous signaling by a regulatory system unique to the pandemic PMEN1 pneumococcal lineage
Source: PLoS Pathog. 2017 May 18;13(5):e1006339. doi: 10.1371/journal.ppat.1006339 (PMC5436883; doi:10.1371/journal.ppat.1006339)
Supplement: S5 Table — (PDF) [file ppat.1006339.s008.pdf]

Table S5. List of genes with at least 2 fold difference in expression levels between wild-type PN4595-T23 and the isogenic *tprA2* strain.

Gene ID	Annotation	Fold change (*tprA2*/WT)	*P*-value	Bonferroni	Benjamini-Hochberg
SPN23F_12701	*lcpA*	62.11	0	0	0
CGSSp4595_1258	putative ABC transporter, permease protein	58.08	0	0	0
CGSSp4595_1257	*lcpM*	45.39	0	0	0
CGSSp4595_1256	*lcpT*	40.38	0	0	0
CGSSp4595_1260	ABC transporter, ATPase	34.70	0	0	0
CGSSp4595_1255	FIG01114468: hypothetical protein	33.68	0	0	0
CGSSp4595_1261	*phrA2*	32.56	0	0	0
CGSSp4595_1259	hypothetical protein	31.72	0	0	0
CGSSp4595_1253	unknown	31.62	0	0	0
CGSSp4595_1254	FIG01116415: hypothetical protein	28.36	0	0	0
CGSSp4595_1267	SNF2 family protein	6.99	0	0	0
CGSSp4595_1263	FIG01118149: hypothetical protein	6.69	0	0	0
CGSSp4595_1266	Retron-type RNA-directed DNA polymerase (EC 2.7.7.49)	4.48	1.06E-13	2.04E-10	9.71E-12
CGSSp4595_1264	unknown	4.24	0	0	0
CGSSp4595_1270	conserved domain protein	2.76	1.89E-06	0.0036369	5.87E-05
CGSSp4595_0698	hypothetical protein	2.28	0.00012	0.2259078	0.003094627
CGSSp4595_1274	FIG01114970: hypothetical protein	2.27	4.82E-05	0.0930484	0.001348527
CGSSp4595_1698	Transcriptional regulator, GntR family	2.24	3.28E-10	6.32E-07	1.41E-08
CGSSp4595_1552	FIG01114360: hypothetical protein	2.21	9.73E-12	1.88E-08	5.69E-10
CGSSp4595_1549	DNA-cytosine methyltransferase (EC 2.1.1.37)	2.20	2.88E-11	5.56E-08	1.54E-09
CGSSp4595_0276	Transposase of IS657	2.18	0.00022	0.4150853	0.005321607
CGSSp4595_0297	putative ATP-dependent Clp proteinase (ATP-binding subunit)	2.11	1.05E-05	0.0201752	0.000296694
CGSSp4595_0367	Macrophage infectivity potentiator protein	2.07	5.61E-07	0.0010831	1.84E-05
CGSSp4595_1674	probable beta-D-galactosidase	2.04	0.00273	1	0.039885863
CGSSp4595_1273	Tn5252, Orf23	2.03	0.00128	1	0.02271382
CGSSp4595_1531	putative large terminase subunit	2.02	1.12E-10	2.16E-07	5.14E-09
CGSSp4595_1550	Single-stranded DNA-binding protein	2.01	3.13E-11	6.05E-08	1.59E-09
CGSSp4595_0800	Type I restriction-modification system, specificity subunit S (EC 3.1.21.3)	-4.23	0	0	0
CGSSp4595_1262	transcriptional regulator, *tprA2*	-18.10	0	0	0
